# Tracing pistachio nuts’ origin and irrigation practices through hyperspectral imaging

**DOI:** 10.1016/j.crfs.2024.100835

**Published:** 2024-09-05

**Authors:** Raquel Martínez-Peña, Salvador Castillo-Gironés, Sara Álvarez, Sergio Vélez

**Affiliations:** a Woody Crops Department, Regional Institute of Agri-Food and Forestry Research and Development of Castilla-La Mancha (IRIAF), Agroenvironmental Research Center “El Chaparrillo”, CM412 Ctra.Porzuna km.4, 13005, Ciudad Real, Spain; b Agroenineering Department, Valencian Institute for Agricultural Research (IVIA), CV-315, km 10.7, 46113, Moncada, Valencia, Spain; c Instituto Tecnológico Agrario de Castilla y León (ITACyL), Ctra. Burgos km 119, 47071, Valladolid, Spain; d Group Agrivoltaics, Fraunhofer Institute for Solar Energy Systems ISE, 79110, Freiburg, Germany

**Keywords:** Geographical location, Hyperspectral imaging, Irrigation treatments, *Pistacia vera*, Traceability

## Abstract

Pistachio trees have become a significant global agricultural commodity because their nuts are renowned for their unique flavour and numerous health benefits, contributing to their high demand worldwide. This study explores the application of Hyperspectral Imaging (HSI) and Machine Learning (ML) to determine pistachio nuts' geographic origin and irrigation practices, alongside predicting essential commercial quality and yield parameters. The study was conducted in two Spanish orchards and employed HSI technology to capture spectral data. It used ML models like Partial Least Squares (PLS), Support Vector Machine (SVM), and Extreme Gradient Boosting (XGBoost) for analysis.

The results demonstrated high accuracy in classifying pistachios based on origin, with accuracies exceeding 94%, and in assessing water content and colour pigments, where both PLS and SVM models achieved 99% accuracy. The research highlighted distinct spectral signatures associated with different irrigation treatments, particularly in the Near-Infrared (NIR) region, with PLS showing an accuracy of 92%. However, challenges were noted in predicting fruit orientation, while predicting height location within the tree was more successful, reflecting clearer spectral distinctions. Regression models also showed promise, particularly in predicting yield (R^2^ = 0.89 with PLS) and percentage of blank nuts (R^2^ = 0.71 with PLS). The correlation analysis revealed key insights, such as an inverse relationship between blank nuts and yield, and a strong correlation between yield and split nuts. Despite challenges in predicting fruit orientation, the research showed promising results in forecasting yield and commercial quality factors, indicating the effectiveness of spectral analysis in optimising pistachio production and sustainability.

## Highlights

• Hyperspectral Imaging (HSI) and Machine Learning (ML) are useful for pistachio traceability.
• Differentiated pistachios' geographic origin with over 94% F1 scores.
• Identified irrigation treatment impacts via spectral signatures at 970 nm.
• Achieved high accuracy in predicting pistachio yield and quality factors.
• Demonstrated HSI's potential in sustainable precision agriculture.

## Graphical abstract

**Figure:**
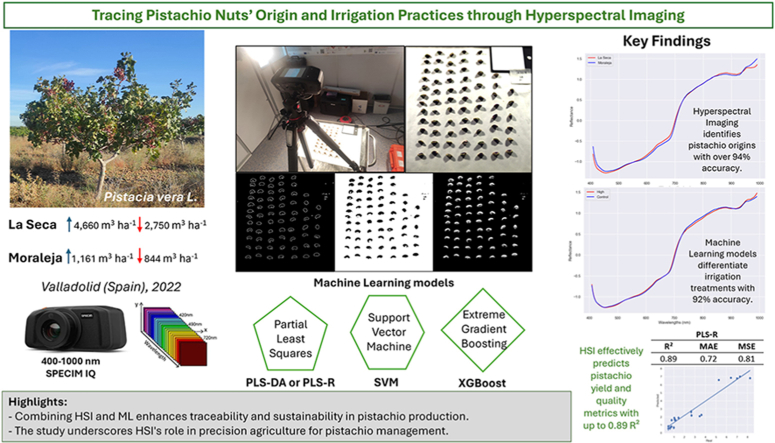

## 1 Introduction

Pistachio trees, originating from southern Central Asia, have expanded globally, with evidence suggesting their westward spread to areas like modern Syria at least 2000 years ago (Mir-Makhamad et al., 2022). Pistachio nuts, known worldwide for their distinctive flavour and use in different cuisines, have attracted attention for their numerous health benefits, such as high protein, dietary fibre, and essential vitamins and minerals (Mandalari et al., 2021). Pistachios are a nut of great importance for global agriculture, and they have consolidated as profitable crops with growing market demand. In 2022, Iran had the most significant area harvested for pistachios, with 497,484 ha; Turkey came second, with 408,709 ha, and the United States was third, with 173,207 ha (FAO, 2024). However, the production quantity by country in 2022 was reversed, with the United States producing 400,070 t, Turkey producing 241,669 t, and Iran producing 239,289 t. Spain has seen a growing interest in cultivating pistachios lately, and even though the market experienced a decline in 2022, the Spanish pistachio market has demonstrated its strength over time with a significant rise in consumption despite dealing with issues of market instability and competition from imports from other countries (CBI - Centre for the Promotion of Imports from developing countries, 2020).

The food industry faces challenges like food fraud (Meerza et al., 2019). Specifically, the pistachio industry deals with issues such as mislabelling regarding origin and adulteration (Aykas and Menevseoglu, 2021). In addition, irrigation practices can affect pistachio nut quality (Martínez-Peña et al., 2023). These problems require developing robust, non-invasive, and efficient techniques to trace their geographical origin and determine pistachio irrigation treatments. Spectroscopy, analysing the electromagnetic radiation of an object to identify composition and properties, emerges as a solution. It involves measuring the spectrum of light absorbed, emitted, or scattered by materials and it has been used in the agri-food production sector to evaluate fruit quality (Lin and Ying, 2009), leaf water content (Rodríguez-Pérez, 2017), and disease assessment (Vélez et al., 2024; Xie et al., 2017). Hyperspectral Imaging (HSI), which integrates the advantages of imaging systems and spectroscopic instruments to provide spatially resolved spectral data (Wu et al., 2022), has also been widely used in the agri-food sector.

In contrast to traditional spectrometers' capabilities, HSI captures spectral profiles across areas rather than mere points, offering a comprehensive characterisation of absorption and reflection bands linked to objects and their prevailing conditions (Khan et al., 2022). It has demonstrated its feasibility for disease detection, classification, grading and detection of chemical attributes among various agricultural products (B. Wang et al., 2023). The principle of HSI consists of capturing and analysing images across a broad spectrum of wavelengths, offering a systematic approach to detect nuances in agricultural products, including grains, fruits, vegetables and meats (Zhu et al., 2020). During the last two decades, HSI has been widely researched, showing its promising potential for measuring quality and protecting horticultural and agricultural products. Its evolution from remote sensing, computer vision, and point spectroscopy provides superior image segmentation for defect and contamination detection, thus ensuring the quality of agricultural products and facilitating the extraction of relevant spectral signatures (Sethy et al., 2022). These signatures are vital in acquiring crucial agricultural information and detecting both external and internal quality attributes of farming products, such as pistachio nuts (C. Wang et al., 2021). The post-harvest phase is particularly critical, as it is laden with biosecurity, diagnostics, and quality assessment challenges, all of which significantly impact the product's commercial value and consumer acceptance (Palumbo et al., 2022).

Nevertheless, unlike RGB images that consist of three colour channels, HSI usually contains hundreds of spectral bands. While exploiting this wealth of information is complicated, its potential is undeniable, and new techniques are continually evolving to analyse this data better (L. Wang and Zhao, 2016). In this sense, Machine Learning (ML) techniques have facilitated and boosted the application of HSI as non-destructive, real-time techniques for assessing food quality and safety within the supply chain, from sorting to sales (Kang et al., 2022). Thus, integrating HSI with ML methodologies has revolutionised non-destructive testing across various sectors, especially in agriculture and food quality assessment. ML has opened the door to estimating pistachio mass precisely, highlighting the efficacy of integrating spectral data for enhanced agricultural produce evaluation (Saglam and Cetin, 2022), detecting damage in mango using NIR hyperspectral images, not visible to the naked eye (Vélez Rivera et al., 2014), accurately predict internal quality parameters of apples (Çetin et al., 2022), and designing specialised sensing systems via spectral filters for mango ripeness estimation through field hyperspectral imaging and ML (Gutiérrez et al., 2019). These advances demonstrate the significant impact of the fusion of HSI and ML in advancing food quality, improving agri-food production and safety inspection, and promoting a shift towards more efficient, accurate and non-destructive methodologies.

This study explores the potential of HSI technology and Machine Learning to discriminate between different irrigation treatments and geographical origins of pistachio nuts harvested from two orchards in Spain. Employing Python, libraries like Scikit and ML models such as PLS-DA, PLS-R, SVM, and XGBoost, this study hypothesises that different irrigation methods and locations significantly affect pistachio yield and commercial quality, and these effects can be identified using HSI technology. HSI images were used to build models to classify pistachios based on their origin and irrigation treatments, improving their traceability and authenticity.

## 2 Materials and methods

### 2.1 Study site and plant material

In 2022, experiments were performed in two pistachio orchards located in Valladolid, Castilla y León, Spain. These orchards were situated in “Moraleja de las Panaderas” (M) (X: 347,802.062; Y: 4,570,373.104) and “La Seca” (S) (X: 341,455.662; Y: 4,589,735.763), areas in the southern part of the province.

The plant material selected for the experiments consisted of 7-year-old (Moraleja) and 15-year-old (La Seca) pistachio plants from the *Pistacia vera* cv. Kerman variety. This variety, one of the prevalent female cultivars in Spain, is esteemed for its exceptional nut quality and adaptability to diverse environmental conditions (Mandalari et al., 2021). These plants were grafted onto the UCB rootstock, a *P. atlantica × P. integerrima hybrid*. The orchard design adopted a 7 × 6 m triangular planting pattern with a NE–SW orientation, maximising sunlight interception and ensuring efficient resource utilisation. The male cultivar used in these orchards was cv. Peter. Standard agricultural practices were followed, including applying specific agrochemicals to manage weeds, pests, and diseases in order to safeguard yield potential.

### 2.2 Irrigation treatments

**Fig. 1:**
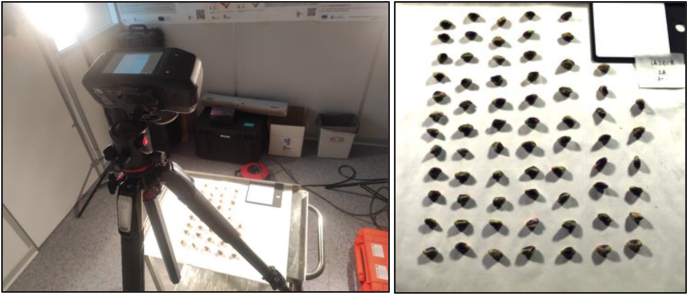
Setup of the SPECIM IQ hyperspectral camera on a tripod to capture images during indoor measurements.

The pistachio trees (Fig. 1) were exposed to two distinctive irrigation treatments during their growth phase. The high irrigation treatment (H) delivered 50% more water than the control treatment (C). In “La Seca”, trees were irrigated from January to October 2022 using a computer-controlled drip irrigation system. The duration of each irrigation episode was used to vary the amount of water applied in each treatment, depending on the season and climatic conditions. In 2022, trees at “La Seca” received total irrigation volumes of 2750 m^3^ ha^−1^ for the control treatment (SC) and 4660 m^3^ ha^−1^ for the high irrigation treatment (SH).

Conversely, in “Moraleja”, irrigation was provided from May to October. The control treatment (MC) received 844 m^3^ ha^−1^, while the high treatment (MH) was allocated 1161 m^3^ ha^−1^. Such systematic variation in water provisioning was executed to study the impacts of differential irrigation on pistachio tree growth and productivity.

### 2.3 Harvest assessment and commercial quality

Upon reaching maturity in October 2022, twenty trees, evenly split between the two irrigation treatments and locations (five replicates per condition), were harvested. Agronomic and commercial quality metrics were gauged after harvesting. The yield was estimated with post-harvest samples that underwent peeling, dried for 24 h at 60 °C to mitigate mycotoxin risks and weighed (Yield, kg tree^−1^). The size was determined by the number of pistachios in one ounce (Commercial Caliber, 28.35 g). The percentage of open husk (Split), closed husk (Non-Split), and empty nuts (Blank) were calculated per tree at a representative subsample of twenty five nuts from the yield obtained, without taking into account the height and orientation of the fruit on the tree.

### 2.4 Image capturing

Hyperspectral imagery was captured using the SPECIM IQ camera (SPECIM IQ camera, Specim Spectral Imaging Oy Ltd, Oulu, Finland). It is equipped with a VNIR CMOS sensor, offering a 400–1000 nm spectral range. It features a viewfinder camera with a 5 Mpix resolution, rescaled to 1280*960 pixels for image previews. Specim's proprietary software interface facilitated control and operation. Data is stored on SD cards, with a maximum capacity of 32 GB, in a format that includes Specim Dataset files, compatible with ENVI software. The device operates on a 5200 mAh Li-Ion battery, allowing for approximately 100 measurements per charge and storage cycle. It has a 4.3-inch touchscreen and 13 physical buttons for user interaction. Connectivity options include USB Type-C and WiFi. The camera's dimensions are 207 x 91 × 74 mm, weighing 1.3 kg. It has an F/number of 1.7, spectral resolution FWHM of 7 nm, and captures data in 204 spectral bands. The peak signal-to-noise ratio exceeds 400:1. For this research, the camera was used within the optimal parameters at temperatures between +5 °C and +40 °C and up to 95% non-condensing humidity.

### 2.5 Image processing and data extraction

The pistachios in every image corresponded to a specific sample collected before harvest, with correspond to three bunches taken from the 20 trees selected in the different locations, according to orientation (North, N; South, S; East, E and West, W) and height (High, H and Low, L). However, due to the age of the trees at the Moraleja location, some samples could not be obtained in the upper or lower part of the trees. After processing, peeling, and drying the samples at 60 °C (24h), a total of 158 images, which corresponded with 2818 pistachios, were obtained. From La Seca, 80 photos were taken (40 of them from the high irrigation treatment and 40 of the control irrigation), and from Moraleja, 77 (37 of them from high irrigation treatment and 40 of the control irrigation).

Python 3.9, along with several external libraries, was used to process images and create models. *Pandas* and *Numpy* were used to work with the data. *Scikit-learn* was used to create the models, while *Scikit-image* and *numpy* were used for image processing. Finally*, seaborn* and *matplotlib* were used for plotting. The reflectance of the images was corrected using white (Spectralon, NH, USA) and dark references. The image correction was done according to the following formula (Sun, 2010), where ${Im}_{Dark}$ and ${Im}_{White}$ are the pixelwise average of the white and dark references, and ${Im}_{RAW}$ is the original image.

(1) $${Im}_{Refl}=\frac{{Im}_{RAW}-{Im}_{Dark}}{{Im}_{White}-{Im}_{Dark}}$$

The number of pistachios in each image varied depending on the tree, treatment and location. The mean spectra of each pistachio were extracted to analyse the images, and Scikitimage's Otsu's binarisation (Otsu, 1979) was used to remove the background. The spectra were scatter-corrected using standard normal variate (SNV) before analysis.

### 2.6 Model generation

ML models were trained using the extracted mean spectra of each pistachio to predict the desired parameters: origin, irrigation treatment, a combination of origin and treatment, fruit orientation in the tree, fruit height in the tree, yield, split, non-split, blank and calibre. Only one predictive value was obtained per image, meaning all pistachios from the same image have the same value for yield, split, non-split, blank and calibre predictions. In addition, a Pearson correlation matrix between the different parameters was also carried out to observe the possible correlations between the data obtained.

Three different commonly used ML models were used: Partial Least Squares Discriminant Analysis (PLS-DA) or regression (PLS-R), Support Vector Machine (SVM), and Extreme Gradient Boosting (XGBoost). A 14-core processor (Intel i9 12th gen; Intel Inc., Santa Clara, CA, USA), 16 GB of RAM DDR5, and an 8 GB GPU (NVIDIA GeForce RTX 3070Ti GPU; Nvidia Inc., Santa Clara, CA, USA) were used for training the models and doing all data analysis.

During the model evaluation, in classification models, accuracy, together with the confusion matrices, show the visualisation of the performance of an algorithm. Each row represents the actual classes, and each column represents the instances of the predicted classes. Accuracy is the ratio of correct predictions to the total number of predictions made by a model. It measures how often the model's predictions are correct overall.

R^2^, Mean Absolute Error (MAE) and Mean Squared Error (MSE) were used to evaluate performance in regression models. R^2^ measures the proportion of the variance in the dependent variable explained by the independent variables in a regression model, MAE measures the average absolute difference between the predicted values and the actual values, whereas MSE measures the average of the squared differences between predicted and actual values.

Before training the models, the dataset, composed of 2819 spectra was randomly divided into a training set to train the models (70% of the spectra, 1973 spectra) and an independent set for validating them (30% of the spectra, 846 spectra) using Scikitlearn's train_test_split function. Then, Scikitlearn's RandomizedsearchCV was used to optimise each model's parameters to predict with the highest accuracy and lowest Root Mean Squared Error (RMSE) using 10-fold cross-validation (Table 1, Table 2).

**Table 1:** Classification models.

Classification models hyperparameters						
Models	PLS-DA		XGBoost		SVM	
**Origin**	Components:	20	Min_child_weight:	5	Kernel:	Linear
			Max_depth:	6	Gamma:	0.001
			Learning_rate:	0.25	Class weight:	Balanced
			Gamma:	0.2	C:	10
			Colsample_bytree:	0.3		
**Irrigation treatment**	Components:	13	Min_child_weight:	5	Kernel:	rbf^a^
			Max_depth:	12	Gamma:	100
			Learning_rate:	0.3	Class weight:	Balanced
			Gamma:	0.2	C:	6.5
			Colsample_bytree:	0.5		
**Origin and Irrigation treatment**	Components:	18	Min_child_weight:	1	Kernel:	Linear
			Max_depth:	4	Gamma:	0.001
			Learning_rate:	0.3	Class weight:	Balanced
			Gamma:	0.1	C:	10
			Colsample_bytree:	0.4		
**Fruit height location**	Components:	14	Min_child_weight:	3	Kernel:	Linear
			Max_depth:	6	Gamma:	0.001
			Learning_rate:	0.25	Class weight:	Balanced
			Gamma:	0.4	C:	8.7
			Colsample_bytree:	0.5		
**Fruit height location La Seca**	Components:	14	Min_child_weight:	5	Kernel:	Linear
			Max_depth:	8	Gamma:	0.001
			Learning_rate:	0.15	Class weight:	Balanced
			Gamma:	0.1	C:	10
			Colsample_bytree:	0.7		
**Fruit height location Moraleja**	Components:	15	Min_child_weight:	5	Kernel:	rbf^a^
			Max_depth:	6	Gamma:	100
			Learning_rate:	0.15	Class weight:	Balanced
			Gamma:	0.0	C:	6.5
			Colsample_bytree:	0.3		
**Fruit height location La Seca control**	Components:	9	Min_child_weight:	3	Kernel:	Linear
			Max_depth:	8	Gamma:	0.01
			Learning_rate:	0.05	Class weight:	Balanced
			Gamma:	0.2	C:	10
			Colsample_bytree:	0.7		
**Fruit height location La Seca high**	Components:	16	Min_child_weight:	3	Kernel:	rbf^a^
			Max_depth:	15	Gamma:	10
			Learning_rate:	0.15	Class weight:	Balanced
			Gamma:	0.1	C:	2.8
			Colsample_bytree:	0.7		
**Fruit height location Moraleja high**	Components:	12	Min_child_weight:	3	Kernel:	rbf^a^
			Max_depth:	5	Gamma:	1
			Learning_rate:	0.3	Class weight:	Balanced
			Gamma:	00.1	C:	0.3
			Colsample_bytree:	0.3		
**Fruit height location Moraleja control**	Components:	5	Min_child_weight:	1	Kernel:	rbf^a^
			Max_depth:	4	Gamma:	10
			Learning_rate:	0.3	Class weight:	Balanced
			Gamma:	0.2	C:	2.8
			Colsample_bytree:	0.5		
**Fruit orientation in the tree**	Components:	20	Min_child_weight:	1	Kernel:	rbf^a^
			Max_depth:	3	Gamma:	10
			Learning_rate:	0.25	Class weight:	Balanced
			Gamma:	0.1	C:	2.8
			Colsample_bytree:	0.5		
**Fruit orientation in the tree Moraleja**	Components:	17	Min_child_weight:	3	Kernel:	rbf^a^
			Max_depth:	6	Gamma:	10
			Learning_rate:	0.25	Class weight:	Balanced
			Gamma:	0.2	C:	4.9
			Colsample_bytree:	0.5		
**Fruit orientation in the tree La Seca**	Components:	18	Min_child_weight:	3	Kernel:	rbf^a^
			Max_depth:	4	Gamma:	10
			Learning_rate:	0.2	Class weight:	Balanced
			Gamma:	0.0	C:	2.8
			Colsample_bytree:	0.3		
**Fruit orientation in the tree La Seca control**	Components:	13	Min_child_weight:	3	Kernel:	Linear
			Max_depth:	4	Gamma:	100
			Learning_rate:	0.1	Class weight:	Balanced
			Gamma:	0.3	C:	0.22
			Colsample_bytree:	0.7		
**Fruit orientation in the tree La Seca high**	Components:	6	Min_child_weight:	1	Kernel:	Linear
			Max_depth:	4	Gamma:	100
			Learning_rate:	0.1	Class weight:	Balanced
			Gamma:	0.3	C:	7.5
			Colsample_bytree:	0.3		
**Fruit orientation in the tree Moraleja high**	Components:	8	Min_child_weight:	3	Kernel:	rbf^a^
			Max_depth:	8	Gamma:	10
			Learning_rate:	0.05	Class weight:	Balanced
			Gamma:	0.2	C:	2.8
			Colsample_bytree:	0.7		
**Fruit orientation in the tree Moraleja control**	Components:	8	Min_child_weight:	7	Kernel:	Linear
			Max_depth:	4	Gamma:	10
			Learning_rate:	0.2	Class weight:	Balanced
			Gamma:	0.0	C:	2.8
			Colsample_bytree:	0.3		

a rbf = Radial Basis Function.

**Table 2:** Regression models.

Regression models hyperparameters						
Models	PLS-R		XGBoost		SVM	
**Yield**	Components:	20	Min_child_weight:	3	Kernel:	Linear
			Max_depth:	3	Gamma:	Scale
			Learning_rate:	0.05	C:	100
			Gamma:	0.4		
			Colsample_bytree:	0.7		
**Split**	Components:	20	Min_child_weight:	3	Kernel:	Poly
			Max_depth:	5	Gamma:	Scale
			Learning_rate:	0.1	C:	500
			Gamma:	0.3		
			Colsample_bytree:	0.3		
**Non-split**	Components:	18	Min_child_weight:	3	Kernel:	Poly
			Max_depth:	5	Gamma:	Scale
			Learning_rate:	0.15	C:	500
			Gamma:	0.01		
			Colsample_bytree:	0.7		
**Blank**	Components:	20	Min_child_weight:	7	Kernel:	Poly
			Max_depth:	5	Gamma:	Scale
			Learning_rate:	0.3	C:	500
			Gamma:	0.2		
			Colsample_bytree:	0.7		
**Calibre**	Components:	19	Min_child_weight:	1	Kernel:	Poly
			Max_depth:	5	Gamma:	Scale
			Learning_rate:	0.05	C:	200
			Gamma:	0.4		
			Colsample_bytree:	0.7		

## 3 Results

### 3.1 Origin

**Fig. 2:**
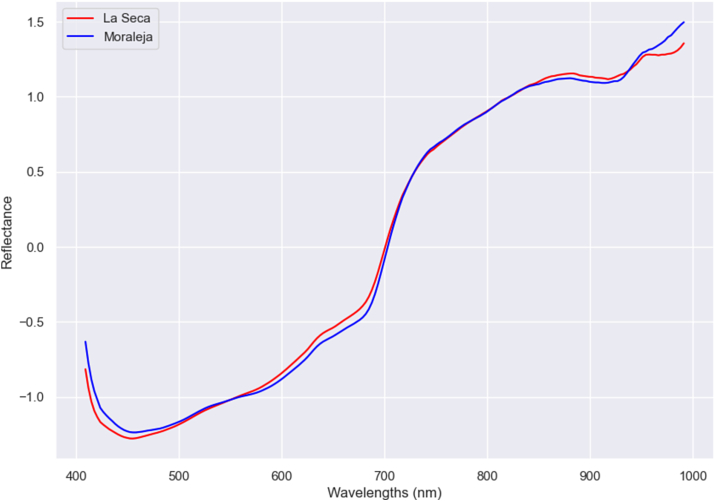
Mean spectra after SNV treatment of La Seca and Moraleja pistachios.

Differences regarding location were found in the mean spectrum, as shown in Fig. 2. Differences appear in the NIR region (970 nm), corresponding with the water content (Büning-Pfaue, 2003). These differences were more pronounced in wavelengths 675 nm (chlorophyll) and 450 nm (carotenoids) (Wellburn, 1994) and at the “La Seca” location. The variation in spectra is clear in the predictions, where all models accuracies above 94%. Both PLS and SVM models performed similarly regarding accuracies (0.99). However, SVM outperformed PLS in predicting La Seca pistachios, while PLS performed better in predicting Moraleja pistachios but worse in predicting La Seca pistachios (Table 3 and Table S1).

**Table 3:** Test set prediction results of the models for pistachio origin classification.

Origin		
PLS	XGBoost	SVM
ACC = 0.99	ACC = 0.94	ACC = 0.99

		Predicted				Predicted				Predicted	
		La Seca	Moraleja			La Seca	Moraleja			La Seca	Moraleja
Real	La Seca	412	3	Real	La Seca	391	24	Real	La Seca	415	0
	Moraleja	5	428		Moraleja	22	409		Moraleja	7	424

**ACC**, Accuracy.

### 3.2 Irrigation treatment

**Fig. 3:**
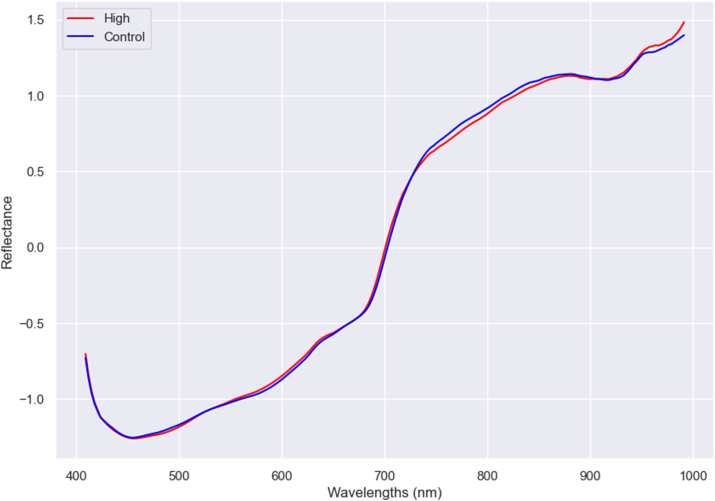
Mean spectra after SNV treatment of the two irrigation treatments (high and control).

Regarding irrigation treatment, some differences were found in the mean spectrum in this study, as shown in Fig. 3. Most differences appear in the peak associated with water (970 nm) in the NIR region (Büning-Pfaue, 2003), which was more pronounced in the La Seca location, and some in wavelengths related to colour pigments such as carotenoids (480 nm). The difference in spectra suggests that the results are correct. Predictions can be made with high accuracies, where XGBoost showed the worst score with a 0.72 value and PLS the best accuracyof 0.92 (Table 4 and Table S2).

**Table 4:** Test set prediction results of the models for pistachio irrigation treatment classification.

Irrigation treatment		
PLS	XGBoost	SVM
ACC = 0.92	ACC = 0.72	ACC = 0.77

Class		Predicted		Class		Predicted		Class		Predicted	
		High	Control			High	Control			High	Control
Real	High	259	59	Real	High	170	148	Real	High	254	64
	Control	9	519		Control	93	435		Control	128	400

**ACC**, Accuracy.

### 3.3 Origin and irrigation treatment

**Fig. 4:**
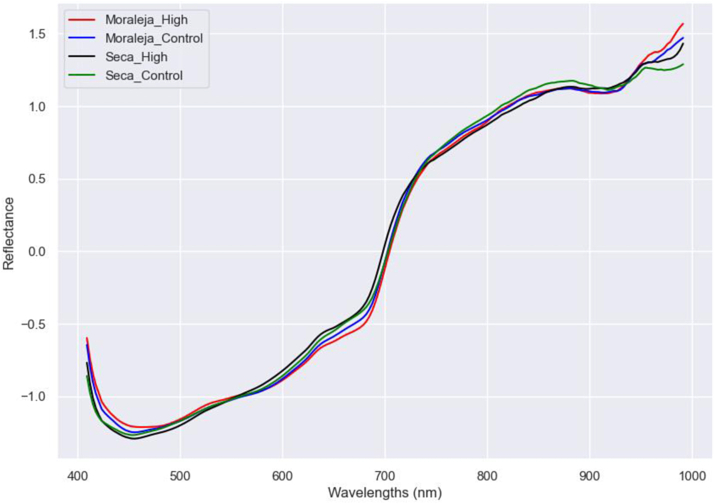
Mean spectra after SNV treatment of each of the origin and irrigation treatment combinations.

Mixing all origins when predicting irrigation treatment can lead to poorer results since it is a more restrictive approach. Therefore, a prediction was made for each irrigation treatment of each origin. Fig. 4 displays significant differences between the four combinations of origin and location across the entire wavelength range. This separation improved classification, resulting in higher accuracies than mixing different location samples. In this study, SVM produced the highest accuracyscore of 0.97, while XGBoost had the lowest, of 0.87 (Table 5 and Table S3).

**Table 5:** Test set prediction results of the models for pistachio origin and irrigation treatment combination classification.

Origin and Irrigation treatment		
PLS	XGBoost	SVM
ACC = 0.95	ACC = 0.87	ACC = 0.97

Class		Predicted				Class		Predicted				Class		Predicted			
		MH	SH	MC	SC			MH	SH	MC	SC			MH	SH	MC	SC
Real	MH	121	0	0	0	Real	MH	90	17	7	7	Real	MH	120	0	1	0
	SH	0	310	0	0		SH	1	296	9	4		SH	0	308	2	0
	MC	0	2	166	29		MC	1	9	161	26		MC	0	0	189	8
	SC	0	0	10	208		SC	2	5	23	188		SC	0	0	15	203

**ACC**, Accuracy**; MH,** Moraleja High; **SH,** La Seca High; **MC,** Moraleja High; **SC,** La Seca control.

### 3.4 Fruit orientation in the tree

**Fig. 5:**
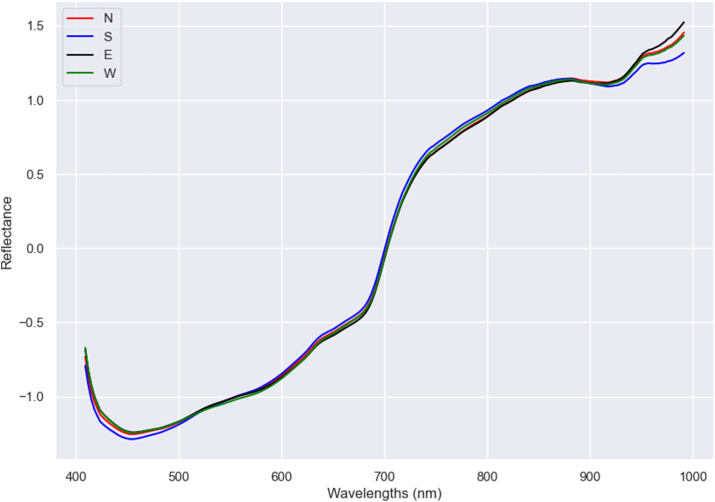
Mean spectra after SNV treatment of each fruit orientation in the three: North (N), South (S), East (E) and West (W).

Climate conditions such as sun, wind, or humidity can vary between each part of the tree. Therefore, orientation was studied and predicted using the models. However, even if Fig. 5 shows some differences in spectra, the prediction of orientation using all data obtained poor accuracies (0.35 for SVM and 0.36 for PLS and XGBoost). Therefore, since predicting from different origins and irrigation treatments with different spectra between them proved to give poor results, the prediction was also made for each origin and each origin and water supply. However, results only increased slightly. When predicting fruit orientation in the tree by origin, the highest accuracyof 0.37 was obtained using SVM for La Seca and with PLS and SVM for Moraleja. In the case of differentiating between origin and irrigation treatment, results increased slightly compared with the ones obtained by separating by origin. In this case, some differences were found regarding the scores: better results were obtained in pistachios with high irrigation treatment (accuracies between 0.36 and 0.5), with XGBoost providing the best results: 0.5 for Moraleja and 0.45 for La Seca. In pistachios with control treatment, results were lower and almost identical for La Seca and Moraleja pistachios: accuracies between 0.36 and 0.39, with PLS providing the best results: 0.38 for Moraleja and 0.39 for La Seca (Table 6 and Table S4). Thus, irrigation treatment seems to influence the differences between the pistachio location and the prediction. No studies were found that tried to predict fruit orientation or location on the tree.

**Table 6:** Test set prediction results of the models for pistachio orientation in the tree classification.

Fruit orientation in the tree		
PLS	XGBoost	SVM
ACC = 0.36	ACC = 0.36	ACC = 0.35

Class		Predicted				Class		Predicted				Class		Predicted			
		N	S	E	W			N	S	E	W			N	S	E	W
Real	N	72	48	29	46	Real	N	72	34	34	55	Real	N	70	32	38	55
	S	57	53	36	67		S	46	69	49	49		S	50	50	68	45
	E	16	61	61	81		E	43	46	78	52		E	41	26	94	58
	W	20	51	33	115		W	42	39	56	82		W	46	39	56	78

Fruit orientation in the tree Moraleja		
PLS	XGBoost	SVM
ACC = 0.36	ACC = 0.36	ACC = 0.37

Class		Predicted				Class		Predicted				Class		Predicted			
		N	S	E	W			N	S	E	W			N	S	E	W
Real	N	74	19	34	0	Real	N	60	19	18	30	Real	N	53	25	19	30
	S	58	31	31	0		S	29	20	35	36		S	30	28	37	25
	E	26	25	56	0		E	18	14	47	28		E	20	17	47	23
	W	29	20	39	0		W	23	11	23	31		W	20	12	19	37

Fruit orientation in the tree La Seca		
PLS	XGBoost	SVM
ACC = 0.37	ACC = 0.36	ACC = 0.37

Class		Predicted				Class		Predicted				Class		Predicted			
		N	S	E	W			N	S	E	W			N	S	E	W
Real	N	14	38	0	32	Real	N	18	20	21	25	Real	N	35	12	14	23
	S	11	54	0	48		S	15	52	23	23		S	25	44	25	19
	E	0	33	0	59		E	7	27	30	28		E	22	20	29	21
	W	0	36	0	80		W	15	28	27	46		W	36	18	20	42

Fruit orientation in the tree Moraleja control		
PLS	XGBoost	SVM
ACC = 0.38	ACC = 0.36	ACC = 0.37

Class		Predicted				Class		Predicted				Class		Predicted			
		N	S	E	W			N	S	E	W			N	S	E	W
Real	N	41	31	10	0	Real	N	41	7	12	22	Real	N	60	14	8	0
	S	21	47	20	0		S	15	19	35	19		S	36	28	21	3
	E	7	35	36	0		E	10	15	30	23		E	23	24	28	3
	W	15	31	29	0		W	18	8	22	27		W	31	20	21	3

Fruit orientation in the tree Moraleja high		
PLS	XGBoost	SVM
ACC = 0.38	ACC = 0.50	ACC = 0.43

Class		Predicted				Class		Predicted				Class		Predicted			
		N	S	E	W			N	S	E	W			N	S	E	W
Real	N	30	0	9	0	Real	N	22	5	9	3	Real	N	22	5	6	6
	S	26	0	6	0		S	10	11	7	4		S	11	9	6	6
	E	12	0	15	0		E	3	2	20	2		E	3	4	16	4
	W	13	0	8	0		W	5	2	8	6		W	11	2	4	4

Fruit orientation in the tree La Seca high		
PLS	XGBoost	SVM
ACC = 0.36	ACC = 0.45	ACC = 0.43

Class		Predicted				Class		Predicted				Class		Predicted			
		N	S	E	W			N	S	E	W			N	S	E	W
Real	N	0	12	0	15	Real	N	2	2	1	22	Real	N	17	0	1	9
	S	0	35	0	33		S	2	33	16	17		S	11	23	20	7
	E	0	18	0	22		E	2	11	17	10		E	12	1	20	7
	W	0	22	0	35		W	7	1	15	34		W	30	0	4	23

Fruit orientation in the tree La Seca control		
PLS	XGBoost	SVM
ACC = 0.39	ACC = 0.38	ACC = 0.37

Class		Predicted				Class		Predicted				Class		Predicted			
		N	S	E	W			N	S	E	W			N	S	E	W
Real	N	17	35	0	4	Real	N	18	11	13	14	Real	N	23	1	19	13
	S	12	53	0	5		S	17	25	13	15		S	16	13	24	17
	E	4	30	0	5		E	5	11	19	4		E	6	1	27	5
	W	2	32	0	14		W	9	14	7	18		W	10	9	13	16

**ACC**, Accuracy; **N**, North; **S**, South; **E**, East; **W,** West.

### 3.5 Fruit height location in the tree

**Fig. 6:**
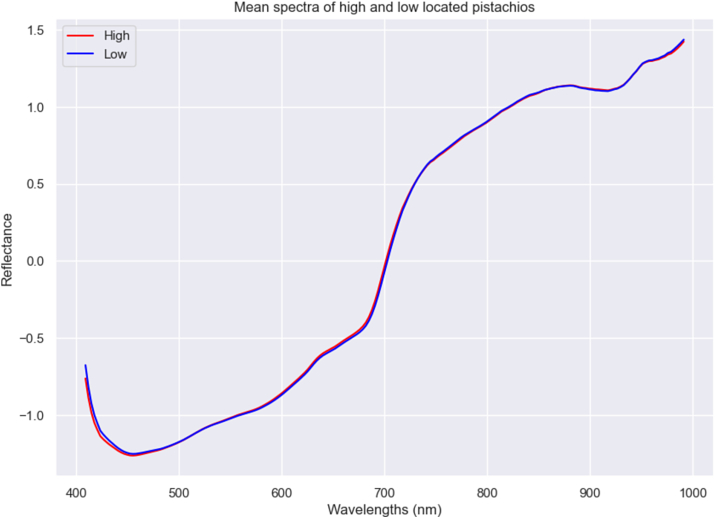
Mean spectra after SNV treatment of each fruit height location in the tree (high and low).

Similarly, as in orientation, there are sun, wind, and humidity differences between high and low pistachios in the tree. For that reason, height location was tried to be studied and predicted. Fig. 6 shows little differences in spectra. However, the prediction of pistachio height location in the tree obtained better results than predicting fruit orientation in the tree. Using all data, accuracies of 0.61 for PLS and 0.59 for XGBoost and SVM were obtained (Table 7 and Table S5). Each prediction was made for fruit orientation in the tree, origin, irrigation treatment, and combination of origin and water treatment. When predicting fruit orientation in the tree by origin, results increased slightly. The highest accuracies were obtained using PLS for La Seca and Moraleja, with accuracies of 0.69 and 0.62, respectively. In the case of differentiating between origin and irrigation treatment, results increased slightly compared with the ones obtained by separating by origin. The same differences as in fruit orientation in the tree prediction were found regarding the scores: better results were obtained in pistachios with high irrigation treatment (accuracies between 0.5 and 0.75), with PLS providing the best results: 0.71 for Moraleja and 0.75 for La Seca. In pistachios with control treatment, results were lower and almost identical for La Seca and Moraleja pistachios: accuracies between 0.54 and 0.64, with PLS providing the best results: 0.55 for Moraleja and 0.64 for La Seca (Table 7 and Table S5). Thus, it is possible to predict fruit height location on the tree at an origin and irrigation treatment level, but not so well at a higher level due to the difference in spectra between origin and treatment combinations.

**Table 7:** Test set prediction results of the models for pistachio height in the tree classification.

Fruit height in the tree		
PLS	XGBoost	SVM
ACC = 0.61	ACC = 0.59	ACC = 0.59

Class		Predicted				Predicted		Class		Predicted	
		High	Low			High	Low			High	Low
Real	High	306	140	Real	High	281	165	Real	High	272	174
	Low	191	209		Low	178	222		Low	170	230

Fruit orientation in the tree Moraleja		
PLS	XGBoost	SVM
ACC = 0.62	ACC = 0.52	ACC = 0.53

Class		Predicted		Class		Predicted		Class		Predicted	
		High	Low			High	Low			High	Low
Real	High	107	98	Real	High	107	96	Real	High	93	112
	Low	68	169		Low	114	123		Low	96	142

Fruit orientation in the tree La Seca		
PLS	XGBoost	SVM
ACC = 0.69	ACC = 0.62	ACC = 0.57

Class		Predicted		Class		Predicted		Class		Predicted	
		High	Low			High	Low			High	Low
Real	High	210	36	Real	High	164	82	Real	High	110	136
	Low	89	70		Low	73	86		Low	37	122

Fruit orientation in the tree Moraleja control		
PLS	XGBoost	SVM
ACC = 0.55	ACC = 0.54	ACC = 0.60

Class		Predicted		Class		Predicted		Class		Predicted	
		High	Low			High	Low			High	Low
Real	High	86	66	Real	High	87	64	Real	High	81	70
	Low	79	93		Low	84	88		Low	60	112

Fruit orientation in the tree Moraleja high		
PLS	XGBoost	SVM
ACC = 0.71	ACC = 0.50	ACC = 0.58

Class		Predicted		Class		Predicted		Class		Predicted	
		High	Low			High	Low			High	Low
Real	High	34	22	Real	High	22	34	Real	High	20	36
	Low	13	50		Low	26	37		Low	13	50

Fruit orientation in the tree La Seca high		
PLS	XGBoost	SVM
ACC = 0.75	ACC = 0.69	ACC = 0.65

Class		Predicted		Class		Predicted		Class		Predicted	
		High	Low			High	Low			High	Low
Real	High	97	13	Real	High	89	21	Real	High	76	34
	Low	35	47		Low	37	45		Low	33	49

Fruit orientation in the tree La Seca control		
PLS	XGBoost	SVM
ACC = 0.64	ACC = 0.58	ACC = 0.58

Class		Predicted		Class		Predicted		Class		Predicted	
		High	Low			High	Low			High	Low
Real	High	81	36	Real	High	61	56	Real	High	35	82
	Low	41	56		Low	32	64		Low	8	88

**ACC**; Accuracy.

### 3.6 Yield, split, non-split, blank and calibre predictions

Yield, split, non-split, blank and calibre are significant quality factors that play a crucial role in pistachio production. In order to determine whether it was possible to predict these parameters, PLS, XGBoost, and SVM regression models were developed. The results of these models and the test set prediction plots (seaborn regplot) are presented in Table 8 and Table S6.

**Table 8:** Test set prediction results of the regression models for pistachio yield, split, non-split, blank and calibre.

Yield								
PLS			XGBoost			SVM		
R^2^	MAE	MSE	R^2^	MAE	MSE	R^2^	MAE	MSE
0.89	0.72	0.81	0.38	1.84	4.57	0.88	0.72	0.89
		

**Split**								
**PLS**			**XGBoost**			**SVM**		
**R**^**2**^	**MAE**	**MSE**	**R**^**2**^	**MAE**	**MSE**	**R**^**2**^	**MAE**	**MSE**
0.56	6.74	74.15	0.37	8.96	104.33	0.58	6.28	70.98
		

**Non-split**								
**PLS**			**XGBoost**			**SVM**		
**R**^**2**^	**MAE**	**MSE**	**R**^**2**^	**MAE**	**MSE**	**R**^**2**^	**MAE**	**MSE**
0.37	5.30	55.53	0.23	6.13	68.1	0.27	4.97	64.26
		

**Blank**								
**PLS**			**XGBoost**			**SVM**		
**R**^**2**^	**MAE**	**MSE**	**R**^**2**^	**MAE**	**MSE**	**R**^**2**^	**MAE**	**MSE**
**0.71**	**5.11**	49.56	0.48	6.92	90.69	0.67	4.94	57.23
		

**Calibre**								
**PLS**			**XGBoost**			**SVM**		
**R**^**2**^	**MAE**	**MSE**	**R**^**2**^	**MAE**	**MSE**	**R**^**2**^	**MAE**	**MSE**
0.54	0.71	0.75	0.45	0.90	1.21	0.57	0.63	0.70
		

**MAE**, Mean Absolute Error; **MSE**, Mean Squared Error.

Accurately predicting crop yield is crucial for growers as it determines the economic benefits they will receive in the future and the necessary crop inputs. Results of a study show that with the PLS method, a high R^2^ score of 0.89 can be achieved, with SVM an R^2^ value of 0.88, and with XGBoost an R^2^ score of 0.38. Besides, the pistachio split is very important for the farmer. The best prediction result was obtained using SVM, with a maximum R^2^ value of 0.58, R^2^ of 0.56, and PLS and XGBoost produced a result of 0.37 R^2^. Regarding non-split predictions, prediction decreased compared with split prediction, and the highest R^2^ value was obtained using PLS (0.37). However, the results for blank, which is significant for measuring the quality of sellers, were better. The best R^2^ value of 0.71 was obtained using PLS, while the poorest result was obtained using XGBoost, with an R^2^ of 0.48. Regarding calibre, key for the farmer's revenue, the best prediction was obtained using SVM with an R^2^ of 0.57 (Table 8 and Table S6).

### 3.7 Correlation between parameters

**Fig. 7:**
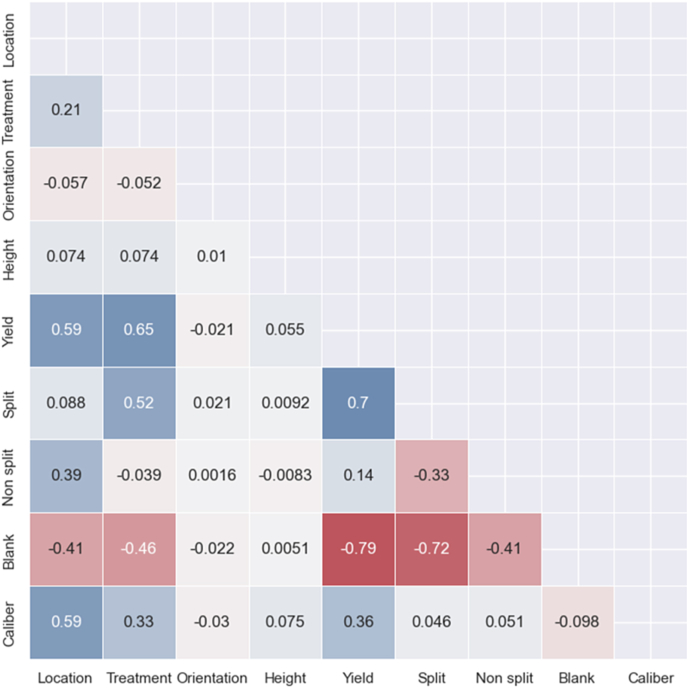
Pearson correlation matrix with all the studied parameters.

Fig. 7 displays the correlations between the yield and commercial quality parameters with the data extracted from the hyperspectral image in a Pearson correlation matrix. The matrix reveals that calibre is related to location (0.59). Moraleja samples have an average bigger calibre (22) than La Seca samples (20), blank was inversely correlated with yield (−0.79), and pistachio split (−0.72). Similarly, the split was highly correlated with yield (0.7) and slightly correlated with treatment (0.52), where control-treated samples showed an average split of 59.2, whereas high-treatment samples showed an average split of 44.9. Additionally, the yield correlated with location (0.59) and irrigation treatment (0.65). The average yield of La Seca samples was 1.9 kg tree^−1^, whereas in Moraleja samples, it was 5.2 kg tree^−1^. Lastly, pistachio height and orientation in the tree did not indicate a correlation with any of the other factors.

## 4 Discussion

This study highlights the applications of non-invasive techniques in pistachio nuts, involving the application of HSI 400–1000 nm to accurately predict the geographic origin of pistachios and evaluate the impact of irrigation practices on their spectral signatures, alongside predicting essential quality and yield metrics. Through rigorous analysis, models employing Partial Least Squares (PLS), Support Vector Machine (SVM), and Extreme Gradient Boosting (XGBoost) demonstrated exceptional capability in distinguishing between pistachios from La Seca and Moraleja, achieving accuracies above 94%. Notably, for water content and colour pigments, both PLS and SVM models reached an accuracy of 0.99, indicating a precise differentiation based on these spectral characteristics. In this sense, the results align with the techniques presented in (Aktaş et al., 2022), where AlexNet and Inception V3 deep learning structures were employed, achieving test accuracies of 96.13% and 96.54%, respectively. Similarly, the application of convolutional neural networks (CNNs) such as AlexNet, VGG16, and VGG19 models demonstrated high success rates in classifying Kirmizi and Siirt pistachio types based on image data, with the VGG16 model achieving the highest classification success of 98.84% (Singh et al., 2022). Furthermore, an improved k-NN classifier, combined with image processing techniques for feature extraction and dimension reduction, also showcased a notable classification success of 94.18% in distinguishing different pistachio species (Ozkan et al., 2021) further demonstrating the successfulness of the deep learning approaches highlighted in this study.

Similarly, (Karadağ and Kılıç, 2023) applied deep learning for object detection, achieving detection accuracies of 98% for open-shelled and 85% for closed-shelled pistachios. Nevertheless, the novelty of the present research is the use of machine learning to identify the Irrigation Treatments and Geographical Origin. In exploring the prediction of pistachios' geographic origin, the results are inspired by those (Soleimanipour et al., 2022), which focus on the cultivar identification of Iranian pistachios using the EfficientNet-B3 model. The study achieved good results, with a hold-out test dataset accuracy of 98.00% and average precision, recall, and accuracy values of 96.73%, 96.70%, and 96.67%, respectively. These outcomes demonstrate the powerful capability of deep learning models in accurately identifying pistachio cultivars, underscoring the technology's importance in ensuring the traceability and authenticity of agricultural products, much like the manuscript's objectives of geographic origin prediction and evaluation of irrigation practices on spectral signatures.

The study's investigation into the effects of irrigation treatment on spectral signatures unveiled significant differences, with high irrigation treatments revealing distinct spectral features, particularly at the 970 nm peak in the Near-Infrared (NIR) region, which is associated with water content (Büning-Pfaue, 2003). This finding is critical, showing spectral data's potential to differentiate irrigation practices. The best accuracy observed for this differentiation was 0.92 with the PLS model, emphasising its efficacy in distinguishing between varying irrigation treatments. Moreover, the differences in the 675 nm and 450 nm bands could be due to colour pigments such as chlorophylls and carotenoids, respectively (Walsh et al., 2020; Wellburn, 1994). The research enhanced classification performance by incorporating geographic origin and irrigation treatment variables. Specifically, combining these factors led to higher accuracies, reaching up to 0.97 with the SVM model. Similar results were found in predicting the origin of *Zanthoxylum bungeanum Maxim* with a 97% accuracy (Ke et al., 2020) or in *Jatropha curcas* L. seeds (Gao et al., 2013) with a 94% correct classification. These findings underscore the subtle impact of both geographical origin and irrigation practices on the spectral signatures of pistachios. The research also revealed that irrigation levels significantly influenced the accuracy of predictions for both fruit orientation and height, with higher irrigation generally improving classification outcomes. This insight could be related to the findings of other authors (Loggenberg et al., 2018; Polder et al., 2024), who found that water influences the hyperspectral response of the plants and emphasised the importance of considering irrigation in spectral analysis for agricultural management. Furthermore, in other crops, other studies did study irrigation treatment, as in waterlogging stress of oilseed rape leaves, with a 96% accuracy. In fruits, up to a 77% accuracy was obtained when discriminating tomato plants under two irrigation regimes (Rinaldi et al., 2015), and in tomato fruits, achieved 100% and 90% accuracy differentiating between water-stressed tomatoes and non-stressed together with abiotic and biotic stress in tomatoes respectively (Susič et al., 2018).

However, the study encountered challenges in predicting the fruit's orientation within the tree, indicated by lower accuracies of 0.35 for SVM and 0.36 for both PLS and XGBoost models. This result reflects the complexity of classifying spectral variances due to fruit orientation. In contrast, predicting the height location of pistachios within the tree was more successful, particularly with PLS models for La Seca and Moraleja, suggesting a clear spectral distinction for pistachios at different heights. These results are paralleled in (Aktaş et al., 2022), where the accuracy difference between industrial and desktop datasets was significant, with desktop dataset training achieving 100% accuracy but dropping to 61.75% when tested with industrial dataset images (Rahimzadeh and Attar, 2022). Also encountered challenges, notably in dealing with occlusions and deformations, yet achieved a counting accuracy of 94.75%. These studies, along with the manuscript's findings, highlight the challenges of ML applications in agriculture, stressing the need for tailored approaches to different problem contexts.

Further, the study developed regression models with high R^2^ scores for predicting yield and commercial quality factors. Specifically, the yield prediction achieved an R^2^ of 0.88 with the SVM model, and the prediction of blank quality reached an R^2^ of 0.71 with the PLS-R model. As previous research (Caporaso et al., 2018; Elmasry et al., 2012; Torres-Rodríguez et al., 2022; B. Wang et al., 2023), these models effectively forecast essential agricultural metrics, offering valuable tools for optimising pistachio production. Therefore, the integration of spectral analysis with precision agriculture technologies could optimise pistachio production and management, a vision shared by (Aktaş et al., 2022), who demonstrated how accurately the industrial dataset performs in classification applications, achieving up to 99.84% accuracy, emphasising the practicality of ML in industrial settings. Similarly, (Karadağ and Kılıç, 2023) introduced a robotic sorting system that significantly enhances the efficiency of sorting processes, offering a glimpse into the future of automated agricultural systems. Both articles, alongside the manuscript, underscore the transformative potential of ML and deep learning technologies in industrial applications, promising improvements in efficiency, productivity, and sustainability within the agricultural sector.

Finally, correlation analysis provided deeper insights, revealing an inverse correlation between blank and yield and a positive correlation between split and yield. This analysis aids in understanding the complex factors influencing pistachio quality and production. Nonetheless, modelling the split and non-split conditions of pistachios proved challenging, with lower R^2^ scores highlighting the difficulties in using spectral data for these specific commercial quality attributes. The relationship between pistachio calibre and geographic location, with a correlation coefficient of 0.59, highlighted the influence of origin on pistachio size, with Moraleja samples typically larger.

This research highlights the significant potential of combining HSI with machine learning in agricultural science, particularly for identifying the unique characteristics of pistachios based on their geographic origin, irrigation methods, and quality markers. Despite challenges in classifying certain features accurately, the findings provide a foundation for future studies to delve into the spectral analysis of diverse pistachio varieties and leverage these insights alongside precision agriculture technologies. Such efforts could markedly improve agricultural productivity and management by examining a broader range of pistachio varieties for distinctive spectral signatures and integrating this knowledge with advanced agricultural practices. The potential application of HSI through drone technology further offers promising avenues for optimising water usage, enhancing crop quality, and promoting sustainable management practices in pistachio orchards. These advancements could ultimately lead to more efficient and sustainable pistachio production, with benefits spanning increased efficiency, enhanced productivity, and reduced environmental impact.

## 5 Conclusions

This study underscores the efficacy of Hyperspectral Imaging (HSI) and Machine Learning (ML) in precisely determining the geographic origin of pistachios, assessing the impact of irrigation practices on their spectral signatures, and forecasting key quality and yield parameters. Utilising advanced modelling techniques such as Partial Least Squares (PLS), Support Vector Machine (SVM), and Extreme Gradient Boosting (XGBoost), we attained high accuracies exceeding 94%, notably in distinguishing pistachios from La Seca and Moraleja based on spectral data. This high level of precision is exemplified by accuracies of 0.99 for both PLS-DA and SVM models in assessing water content and colour pigments, highlighting HSI's potential in precision agriculture.

The research further elucidates the distinct spectral signatures attributable to irrigation treatments, especially the discernible peak at 970 nm in the NIR region, where the PLS model exhibited an accuracy of 0.92. This differentiation capability underscores HSI's application in enhancing sustainable agricultural resource management. However, predicting the orientation of the fruit on the tree presented challenges, contrasting with the more successful prediction of fruit height on the tree, indicating more explicit spectral differences associated with the height of pistachios within the tree.

Additionally, our findings from the correlation matrix reveal that blank quality is inversely proportional to yield and pistachio split, while yield shares a strong correlation with pistachio split and a slight correlation with irrigation treatment. Notably, the location and treatment displayed a low correlation with yield, enriching our understanding of the various factors influencing pistachio commercial quality and yield. Our regression models also showed promising results in predicting yield, with an R^2^ score of 0.88 for yield predictions using the SVM model and 0.71 for blank predictions with the PLS-R model, alongside an R^2^ score of 0.89 for dry matter. These models prove the effectiveness of spectral analysis in forecasting vital agricultural metrics, marking a significant advancement in optimising pistachio production.

Challenges remained in predicting pistachio shell split and calibre, with R^2^ scores of 0.58 and 0.57, respectively, suggesting areas for future improvement. Nevertheless, the study validates HSI as a precise and effective tool for identifying pistachio characteristics based on geographic origin and irrigation practices and predicting commercial quality and yield metrics. The overall findings spotlight the significant potential of spectroscopic analysis in enhancing agricultural practices. Future research should extend to different pistachio varieties and incorporate these insights with cutting-edge agricultural technologies, potentially revolutionising pistachio production management and sustainability.

## CRediT authorship contribution statement

**Raquel Martínez-Peña:** Conceptualization, Methodology, Software, Validation, Formal analysis, Investigation, Resources, Data curation, Writing – original draft, Preparation, Writing – review & editing, Visualization, Supervision. **Salvador Castillo-Gironés:** Methodology, Software, Formal analysis, Investigation, Writing – review & editing. **Sara Álvarez:** Resources, Writing – review & editing, Project administration, Funding acquisition. **Sergio Vélez:** Conceptualization, Methodology, Software, Formal analysis, Data curation, Writing – original draft, Preparation, Writing – review & editing, Supervision.

## Declaration of competing interest

The authors declare that they have no known competing financial interests or personal relationships that could have appeared to influence the work reported in this paper.

## Data Availability

Data will be made available upon reasonable request.
