# Supplementary material for: Tracing pistachio nuts’ origin and irrigation practices through hyperspectral imaging
Source: Curr Res Food Sci. 2024 Sep 5;9:100835. doi: 10.1016/j.crfs.2024.100835 (PMC11414490; doi:10.1016/j.crfs.2024.100835)

**Supplementary table 1.** Train classification results of the models for pistachio origin.

| **Origin** | | | | | | | | | | | |  |
| --- | --- | --- | --- | --- | --- | --- | --- | --- | --- | --- | --- | --- |
| **PLS** | | | | **XGBoost** | | | | **SVM** | | | |  |
| ACC = 1.0 | | | | ACC = 0.99 | | | | ACC = 0.99 | | | |  |
|  | | Predicted | |  | | Predicted | |  | | Predicted | | |
|  |  | La Seca | Moraleja |  |  | La Seca | Moraleja |  |  | La Seca | Moraleja | |
| Real | La Seca | 933 | 0 | Real | La Seca | 933 | 0 | Real | La Seca | 933 | 0 | |
|  | Moraleja | 0 | 1040 |  | Moraleja | 7 | 1033 |  | Moraleja | 3 | 1037 | |

**ACC,** Accuracy

**Supplementary table 2.** Train classification results of the models for pistachio irrigation treatment.

| **Irrigation treatment** | | | | | | | | | | | |
| --- | --- | --- | --- | --- | --- | --- | --- | --- | --- | --- | --- |
| **PLS** | | | | **XGBoost** | | | | **SVM** | | | |
| ACC = 0.93 | | | | ACC = 0.78 | | | | ACC = 0.80 | | | |
| Class | | Predicted | | Class | | Predicted | | Class | | Predicted | |
|  |  | High | Control |  |  | High | Control |  |  | High | Control |
| Real | High | 295 | 22 | Real | High | 548 | 168 | Real | High | 587 | 129 |
|  | Control | 36 | 491 |  | Control | 252 | 1005 |  | Control | 251 | 1006 |

**ACC,** Accuracy

**Supplementary table 3**. Train classification results of the models for origin and irrigation treatment.

| **Origin and Irrigation treatment** | | | | | | | | | | | | | | | | | |
| --- | --- | --- | --- | --- | --- | --- | --- | --- | --- | --- | --- | --- | --- | --- | --- | --- | --- |
| **PLS** | | | | | | **XGBoost** | | | | | | **SVM** | | | | | |
| ACC = 0.96 | | | | | | ACC = 0.95 | | | | | | ACC = 0.99 | | | | | |
| Class | | Predicted | | | | Class | | Predicted | | | | Class | | Predicted | | | |
|  |  | MH | SH | MC | SC |  |  | MH | SH | MC | SC |  |  | MH | SH | MC | SC |
| Real | MH | 271 | 3 | 0 | 0 | Real | MH | 271 | 0 | 1 | 2 | Real | MH | 274 | 0 | 0 | 0 |
|  | SH | 1 | 765 | 0 | 0 |  | SH | 0 | 744 | 13 | 9 |  | SH | 0 | 764 | 1 | 1 |
|  | MC | 0 | 1 | 381 | 60 |  | MC | 0 | 4 | 408 | 30 |  | MC | 0 | 0 | 434 | 8 |
|  | SC | 0 | 0 | 15 | 476 |  | SC | 0 | 2 | 31 | 458 |  | SC | 0 | 1 | 6 | 484 |

**ACC,** Accuracy

**Supplementary table 4.** Train classification results of the models for pistachio orientation.

| **Fruit orientation in the tree** | | | | | | | | | | | | | | | | | |
| --- | --- | --- | --- | --- | --- | --- | --- | --- | --- | --- | --- | --- | --- | --- | --- | --- | --- |
| **PLS** | | | | | | **XGBoost** | | | | | | **SVM** | | | | | |
| ACC = 0.40 | | | | | | ACC = 0.38 | | | | | | ACC = 0.35 | | | | | |
| Class | | Predicted | | | | Class | | Predicted | | | | Class | | Predicted | | | |
|  |  | N | S | E | W |  |  | N | S | E | W |  |  | N | S | E | W |
| Real | N | 235 | 27 | 88 | 126 | Real | N | 201 | 16 | 92 | 167 | Real | N | 190 | 164 | 0 | 122 |
|  | S | 140 | 108 | 128 | 130 |  | S | 120 | 78 | 133 | 175 |  | S | 156 | 161 | 0 | 189 |
|  | E | 124 | 23 | 202 | 134 |  | E | 99 | 15 | 196 | 173 |  | E | 52 | 149 | 0 | 282 |
|  | W | 116 | 24 | 121 | 247 |  | W | 99 | 16 | 124 | 269 |  | W | 55 | 123 | 56 | 330 |
| **Fruit orientation in the tree Moraleja** | | | | | | | | | | | | | | | | | |
| **PLS** | | | | | | **XGBoost** | | | | | | **SVM** | | | | | |
| ACC = 0.39 | | | | | | ACC = 0.38 | | | | | | ACC = 0.37 | | | | | |
| Class | | Predicted | | | | Class | | Predicted | | | | Class | | Predicted | | | |
|  |  | N | S | E | W |  |  | N | S | E | W |  |  | N | S | E | W |
| Real | N | 183 | 0 | 60 | 36 | Real | N | 118 | 64 | 71 | 26 | Real | N | 117 | 65 | 73 | 24 |
|  | S | 99 | 0 | 55 | 58 |  | S | 51 | 92 | 58 | 11 |  | S | 51 | 92 | 58 | 11 |
|  | E | 70 | 0 | 70 | 128 |  | E | 46 | 68 | 131 | 23 |  | E | 46 | 70 | 132 | 20 |
|  | W | 58 | 0 | 62 | 150 |  | W | 67 | 76 | 82 | 45 |  | W | 66 | 76 | 85 | 43 |
| **Fruit orientation in the tree La Seca** | | | | | | | | | | | | | | | | | |
| **PLS** | | | | | | **XGBoost** | | | | | | **SVM** | | | | | |
| ACC = 0.38 | | | | | | ACC = 0.40 | | | | | | ACC = 0.41 | | | | | |
| Class | | Predicted | | | | Class | | Predicted | | | | Class | | Predicted | | | |
|  |  | N | S | E | W |  |  | N | S | E | W |  |  | N | S | E | W |
| Real | N | 34 | 114 | 2 | 31 | Real | N | 51 | 101 | 2 | 29 | Real | N | 71 | 104 | 7 | 37 |
|  | S | 24 | 171 | 1 | 78 |  | S | 23 | 170 | 1 | 80 |  | S | 24 | 169 | 3 | 78 |
|  | E | 6 | 121 | 0 | 108 |  | E | 8 | 119 | 2 | 106 |  | E | 11 | 128 | 19 | 111 |
|  | W | 0 | 93 | 1 | 159 |  | W | 1 | 92 | 3 | 157 |  | W | 2 | 1891 | 4 | 157 |
| **Fruit orientation in the tree Moraleja control** | | | | | | | | | | | | | | | | | |
| **PLS** | | | | | | **XGBoost** | | | | | | **SVM** | | | | | |
| ACC = 0.38 | | | | | | ACC = 0.36 | | | | | | ACC = 0.38 | | | | | |
| Class | | Predicted | | | | Class | | Predicted | | | | Class | | Predicted | | | |
|  |  | N | S | E | W |  |  | N | S | E | W |  |  | N | S | E | W |
| Real | N | 121 | 0 | 81 | 0 | Real | N | 118 | 4 | 80 | 0 | Real | N | 121 | 48 | 33 | 0 |
|  | S | 69 | 0 | 88 | 0 |  | S | 71 | 0 | 86 | 0 |  | S | 48 | 73 | 36 | 0 |
|  | E | 49 | 0 | 160 | 0 |  | E | 49 | 3 | 150 | 7 |  | E | 64 | 57 | 88 | 0 |
|  | W | 55 | 0 | 127 | 3 |  | W | 57 | 0 | 125 | 3 |  | W | 71 | 58 | 54 | 2 |
| **Fruit orientation in the tree Moraleja high** | | | | | | | | | | | | | | | | | |
| **PLS** | | | | | | **XGBoost** | | | | | | **SVM** | | | | | |
| ACC = 0.48 | | | | | | ACC = 0.55 | | | | | | ACC = 0.50 | | | | | |
| Class | | Predicted | | | | Class | | Predicted | | | | Class | | Predicted | | | |
|  |  | N | S | E | W |  |  | N | S | E | W |  |  | N | S | E | W |
| Real | N | 61 | 0 | 1 | 19 | Real | N | 62 | 0 | 1 | 18 | Real | N | 60 | 0 | 4 | 17 |
|  | S | 35 | 0 | 1 | 19 |  | S | 25 | 10 | 3 | 1 |  | S | 25 | 3 | 4 | 7 |
|  | E | 7 | 0 | 21 | 33 |  | E | 9 | 0 | 19 | 33 |  | E | 11 | 0 | 18 | 34 |
|  | W | 13 | 0 | 13 | 51 |  | W | 13 | 0 | 12 | 52 |  | W | 14 | 0 | 13 | 50 |
| **Fruit orientation in the tree La Seca high** | | | | | | | | | | | | | | | | | |
| **PLS** | | | | | | **XGBoost** | | | | | | **SVM** | | | | | |
| ACC = 0.40 | | | | | | ACC = 0.45 | | | | | | ACC = 0.46 | | | | | |
| Class | | Predicted | | | | Class | | Predicted | | | | Class | | Predicted | | | |
|  |  | N | S | E | W |  |  | N | S | E | W |  |  | N | S | E | W |
| Real | N | 0 | 19 | 0 | 46 | Real | N | 16 | 21 | 0 | 38 | Real | N | 51 | 0 | 3 | 11 |
|  | S | 0 | 61 | 0 | 63 |  | S | 0 | 62 | 0 | 62 |  | S | 26 | 46 | 33 | 19 |
|  | E | 0 | 29 | 0 | 88 |  | E | 2 | 28 | 12 | 75 |  | E | 37 | 4 | 49 | 27 |
|  | W | 0 | 23 | 0 | 118 |  | W | 0 | 20 | 5 | 116 |  | W | 62 | 1 | 18 | 60 |
| **Fruit orientation in the tree La Seca control** | | | | | | | | | | | | | | | | | |
| **PLS** | | | | | | **XGBoost** | | | | | | **SVM** | | | | | |
| ACC = 0.39 | | | | | | ACC = 0.41 | | | | | | ACC = 0.37 | | | | | |
| Class | | Predicted | | | | Class | | Predicted | | | | Class | | Predicted | | | |
|  |  | N | S | E | W |  |  | N | S | E | W |  |  | N | S | E | W |
| Real | N | 44 | 10 | 40 | 25 | Real | N | 46 | 10 | 39 | 24 | Real | N | 49 | 4 | 40 | 24 |
|  | S | 23 | 44 | 22 | 36 |  | S | 33 | 49 | 21 | 22 |  | S | 33 | 20 | 49 | 23 |
|  | E | 30 | 12 | 64 | 27 |  | E | 29 | 11 | 65 | 26 |  | E | 27 | 11 | 67 | 26 |
|  | W | 30 | 11 | 38 | 44 |  | W | 28 | 10 | 39 | 46 |  | W | 28 | 8 | 39 | 48 |

**ACC**, Accuracy; **N**, North; **S**, South; **E**, East; **W,** West

**Supplementary table 5**. Train classification results of the models for pistachio height.

| **Fruit height in the tree** | | | | | | | | | | | |
| --- | --- | --- | --- | --- | --- | --- | --- | --- | --- | --- | --- |
| **PLS** | | | | **XGBoost** | | | | **SVM** | | | |
| ACC = 0.63 | | | | ACC = 0.65 | | | | ACC = 0.61 | | | |
| Class | | Predicted | |  | | Predicted | | Class | | Predicted | |
|  |  | High | Low |  |  | High | Low |  |  | High | Low |
| Real | High | 681 | 337 | Real | High | 662 | 356 | Real | High | 626 | 392 |
|  | Low | 390 | 565 |  | Low | 334 | 621 |  | Low | 371 | 584 |
| **Fruit height in the tree Moraleja** | | | | | | | | | | | |
| **PLS** | | | | **XGBoost** | | | | **SVM** | | | |
| ACC = 0.64 | | | | ACC = 0.52 | | | | ACC = 0.60 | | | |
| Class | | Predicted | | Class | | Predicted | | Class | | Predicted | |
|  |  | High | Low |  |  | High | Low |  |  | High | Low |
| Real | High | 321 | 183 | Real | High | 272 | 250 | Real | High | 303 | 201 |
|  | Low | 187 | 335 |  | Low | 241 | 263 |  | Low | 208 | 314 |
| **Fruit height in the tree La Seca** | | | | | | | | | | | |
| **PLS** | | | | **XGBoost** | | | | **SVM** | | | |
| ACC = 0.71 | | | | ACC = 0.67 | | | | ACC = 0.64 | | | |
|  | | | |  | | | |  | | | |
| Class | | Predicted | | Class | | Predicted | | Class | | Predicted | |
|  |  | High | Low |  |  | High | Low |  |  | High | Low |
| Real | High | 383 | 123 | Real | High | 340 | 166 | Real | High | 261 | 245 |
|  | Low | 150 | 287 |  | Low | 144 | 293 |  | Low | 89 | 348 |
| **Fruit height in the tree Moraleja control** | | | | | | | | | | | |
| **PLS** | | | | **XGBoost** | | | | **SVM** | | | |
| ACC = 0.59 | | | | ACC = 0.54 | | | | ACC = 0.60 | | | |
| Class | | Predicted | | Class | | Predicted | | Class | | Predicted | |
|  |  | High | Low |  |  | High | Low |  |  | High | Low |
| Real | High | 242 | 135 | Real | High | 212 | 165 | Real | High | 302 | 75 |
|  | Low | 176 | 200 |  | Low | 86 | 110 |  | Low | 39 | 156 |
| **Fruit height in the tree Moraleja high** | | | | | | | | | | | |
| **PLS** | | | | **XGBoost** | | | | **SVM** | | | |
| ACC = 0.72 | | | | ACC = 0.53 | | | | ACC 0.61 | | | |
| Class | | Predicted | | Class | | Predicted | | Class | | Predicted | |
|  |  | High | Low |  |  | High | Low |  |  | High | Low |
| Real | High | 81 | 47 | Real | High | 68 | 69 | Real | High | 41 | 87 |
|  | Low | 29 | 119 |  | Low | 60 | 78 |  | Low | 20 | 128 |
| **Fruit height in the tree La Seca high** | | | | | | | | | | | |
| **PLS** | | | | **XGBoost** | | | | **SVM** | | | |
| ACC = 0.80 | | | | ACC = 0.73 | | | | ACC = 0.69 | | | |
| Class | | Predicted | | Class | | Predicted | | Class | | Predicted | |
|  |  | High | Low |  |  | High | Low |  |  | High | Low |
| Real | High | 270 | 23 | Real | High | 214 | 79 | Real | High | 203 | 90 |
|  | Low | 65 | 89 |  | Low | 41 | 113 |  | Low | 47 | 106 |
| **Fruit height in the tree La Seca control** | | | | | | | | | | | |
| **PLS** | | | | **XGBoost** | | | | **SVM** | | | |
| ACC = 0.67 | | | | ACC = 0.60 | | | | ACC = 0.64 | | | |
| Class | | Predicted | | Class | | Predicted | | Class | | Predicted | |
|  |  | High | Low |  |  | High | Low |  |  | High | Low |
| Real | High | 121 | 111 | Real | High | 140 | 92 | Real | High | 74 | 158 |
|  | Low | 50 | 214 |  | Low | 10 | 158 |  | Low | 23 | 241 |

**Supplementary table 6**. Train results of the regression models for yield, split, non-split, blank and calibre.

| **Yield** | | | | | | | | |  |
| --- | --- | --- | --- | --- | --- | --- | --- | --- | --- |
| **PLS** | | | **XGBoost** | | | **SVM** | | |  |
| **R²** | **MAE** | **MSE** | **R²** | **MAE** | **MSE** | **R²** | **MAE** | **MSE** |  |
| 0.89 | 1.38 | 2.80 | 0.51 | 2.94 | 11.80 | 0.85 | 1.45 | 3.80 |  |
| 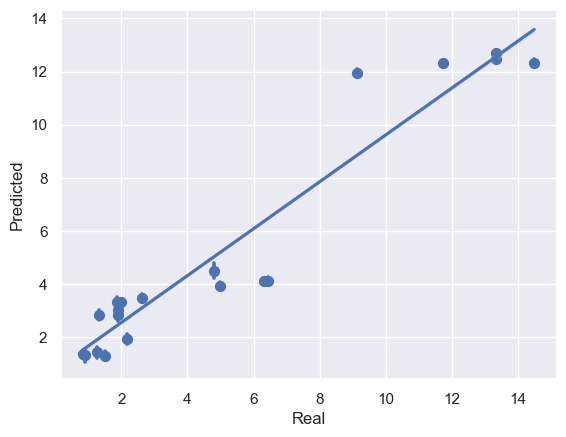 | | | 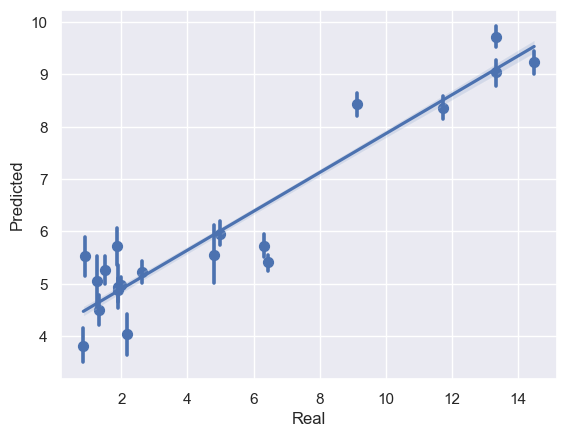 | | | 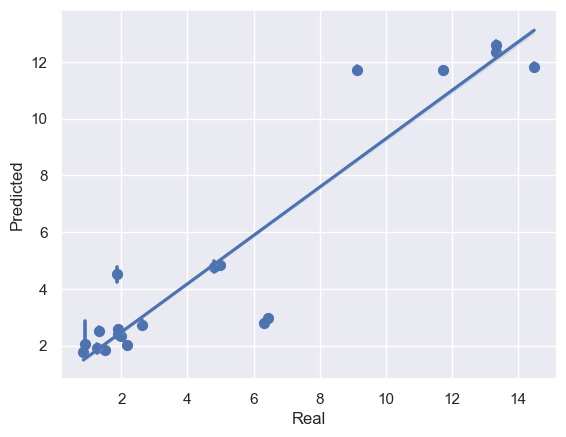 | | |  |
| **Split** | | | | | | | | |  |
| **PLS** | | | **XGBoost** | | | **SVM** | | |  |
| **R²** | **MAE** | **MSE** | **R²** | **MAE** | **MSE** | **R²** | **MAE** | **MSE** |  |
| 0.59 | 6.75 | 75.01 | 0.47 | 7.84 | 86.05 | 0.60 | 6.05 | 70.81 |  |
| 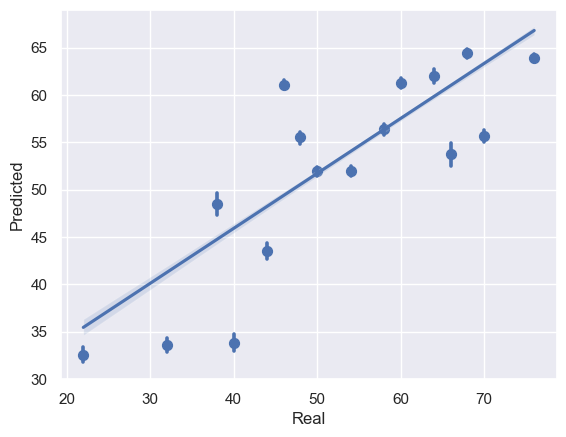 | | | 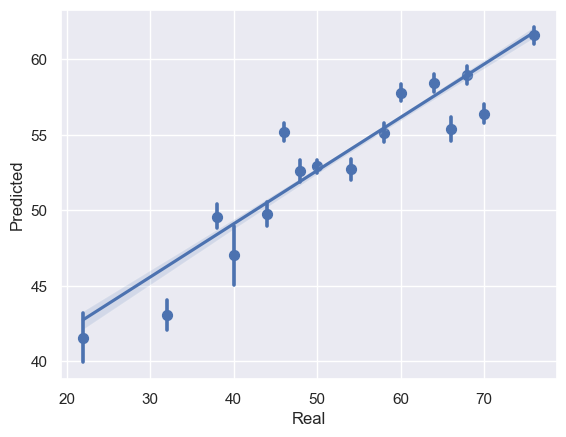 | | | 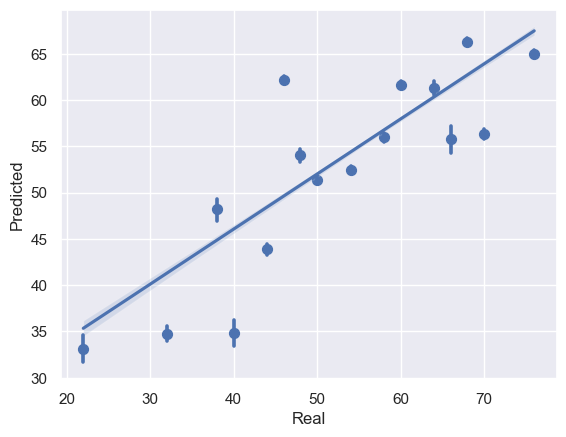 | | |  |
| **Non-split** | | | | | | | | |  |
| **PLS** | | | **XGBoost** | | | **SVM** | | |  |
| **R²** | **MAE** | **MSE** | **R²** | **MAE** | **MSE** | **R²** | **MAE** | **MSE** |  |
| 0.37 | 5.25 | 55.41 | 0.28 | 5.63 | 66.30 | 0.32 | 4.67 | 61.90 |  |
| 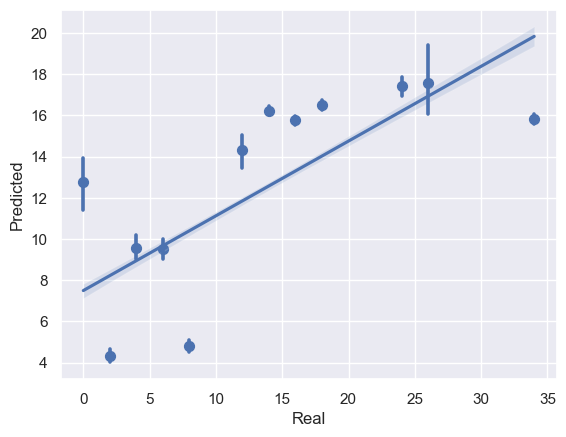 | | | 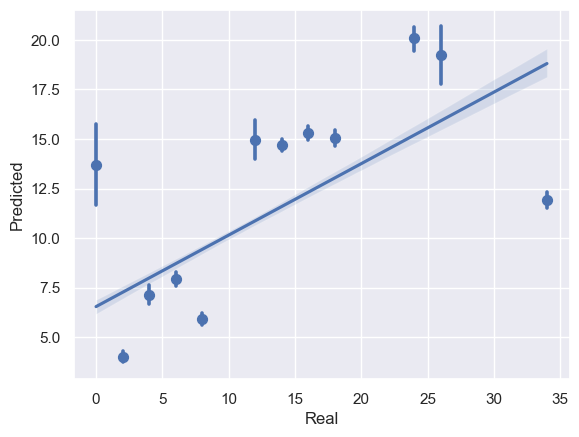 | | | 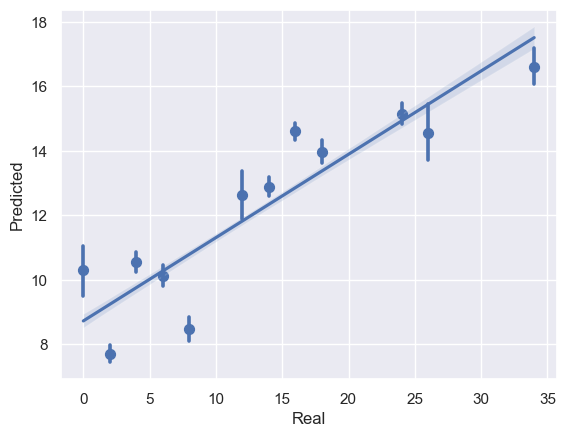 | | |  |
| **Blank** | | | | | | | | |  |
| **PLS** | | | **XGBoost** | | | **SVM** | | |  |
| **R²** | **MAE** | **MSE** | **R²** | **MAE** | **MSE** | **R²** | **MAE** | **MSE** |  |
| 0.75 | 0.89 | 45.15 | 0.67 | 3.42 | 50.69 | 0.72 | 4.53 | 49.20 |  |
| 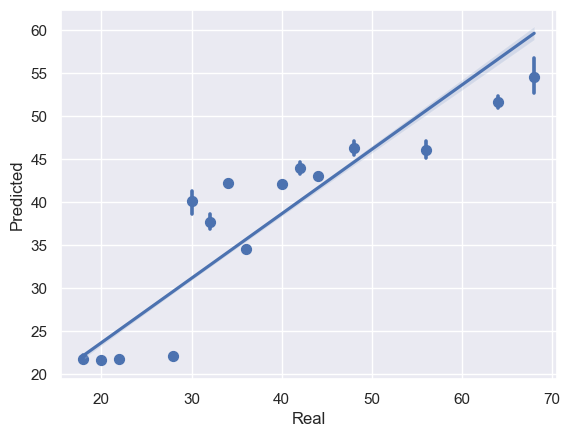 | | | 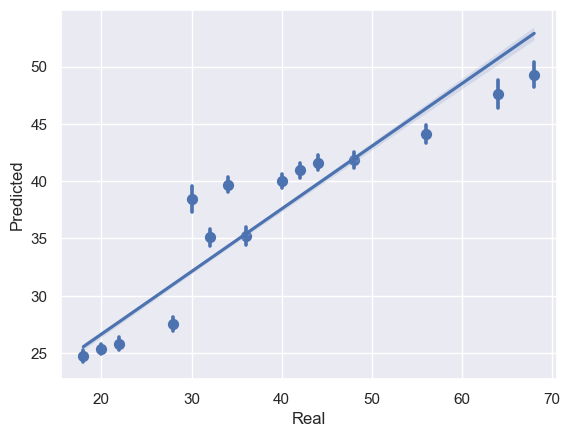 | | | 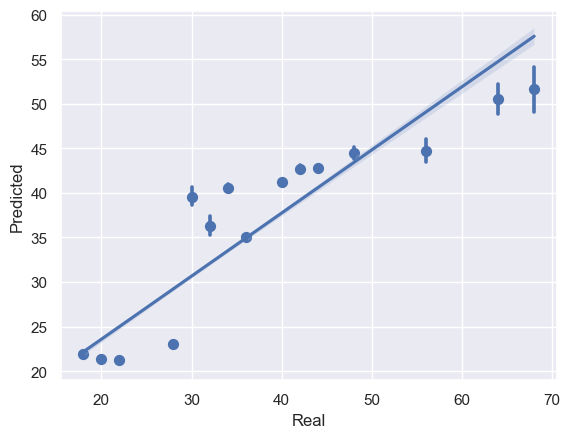 | | |  |
| **Calibre** | | | | | | | | | |
| **PLS** | | | | **XGBoost** | | | **SVM** | | |
| **R²** | | **MAE** | **MSE** | **R²** | **MAE** | **MSE** | **R²** | **MAE** | **MSE** |
| 0.57 | | 0.72 | 0.75 | 0.56 | 0.72 | 0.75 | 0.63 | 0.56 | 0.62 |
| 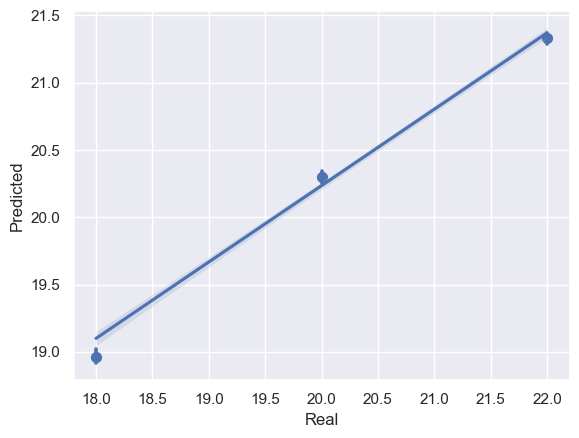 | | | | 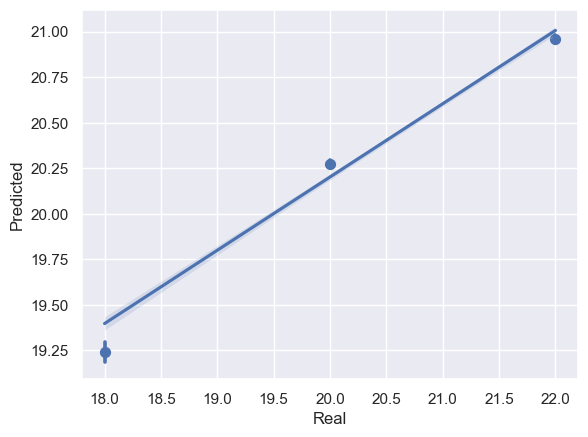 | | | 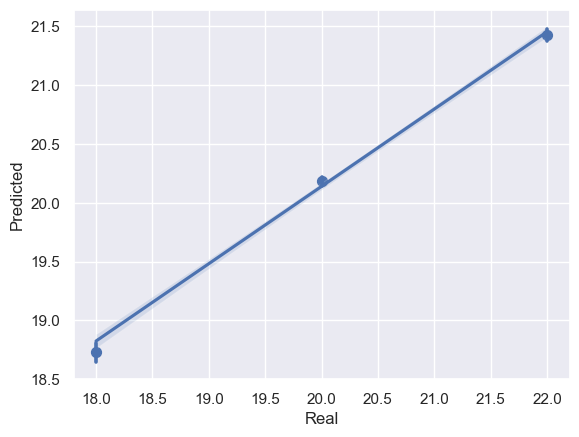 | | |

**MAE**, Mean Absolute Error; **MSE**, Mean Squared Error.

**Supplementary table 7**. PLS VIP SCORES y XGBoost feature importance.


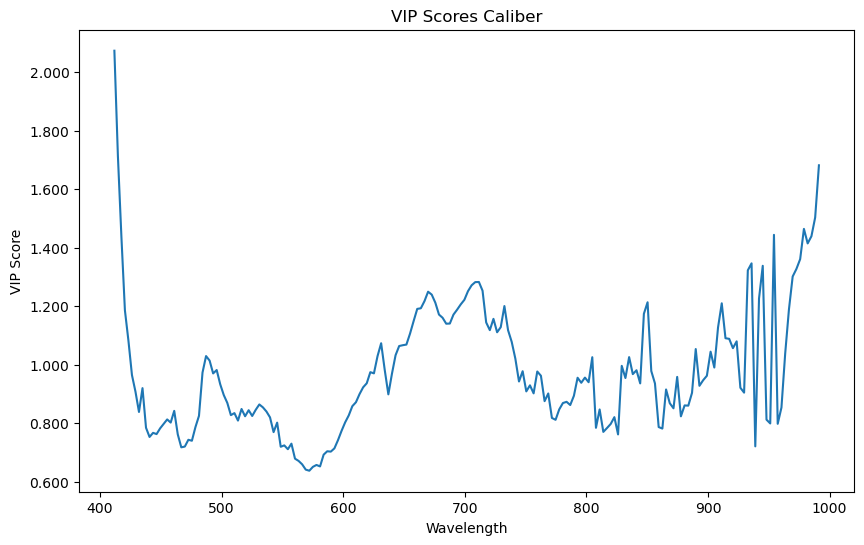

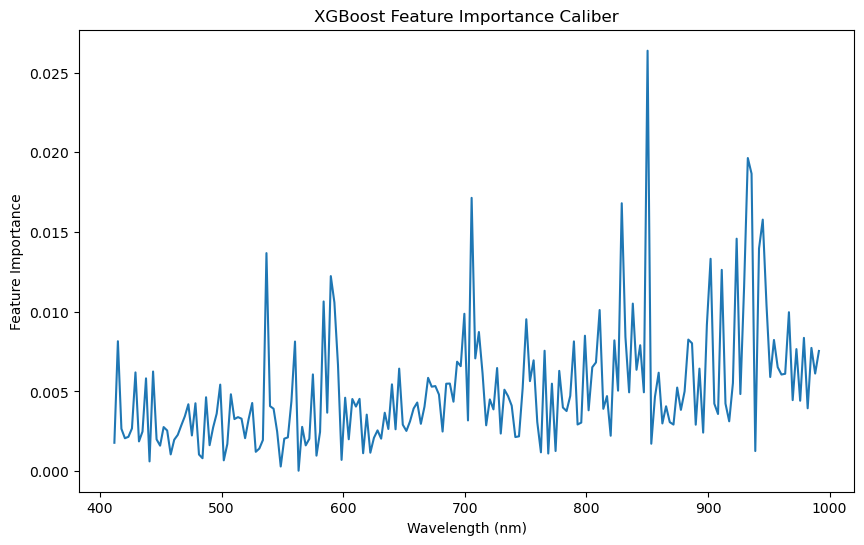


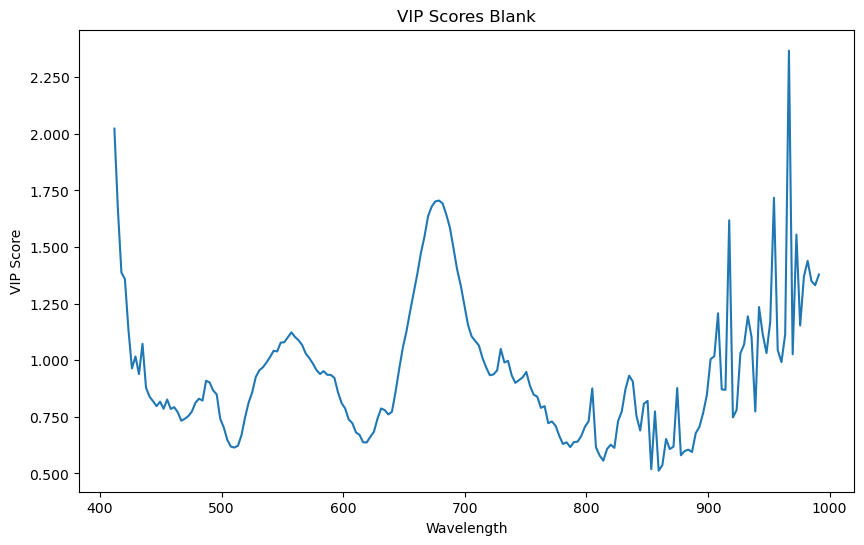

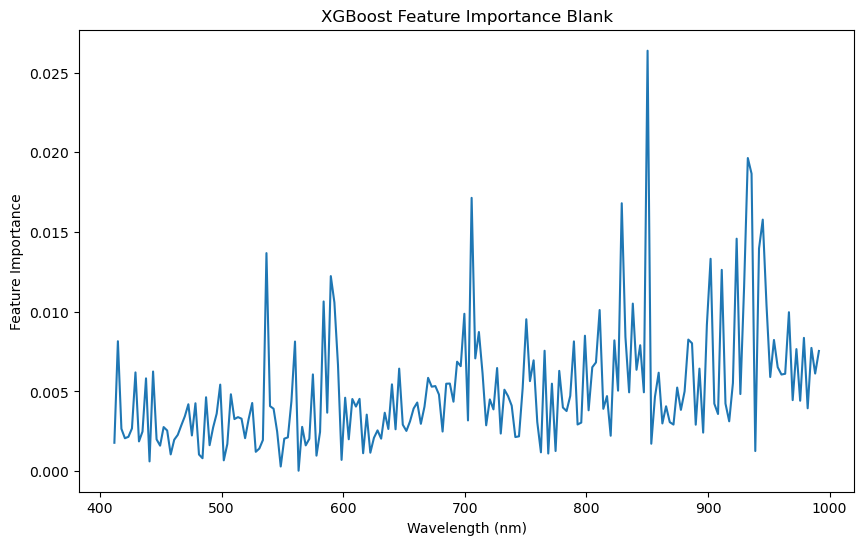


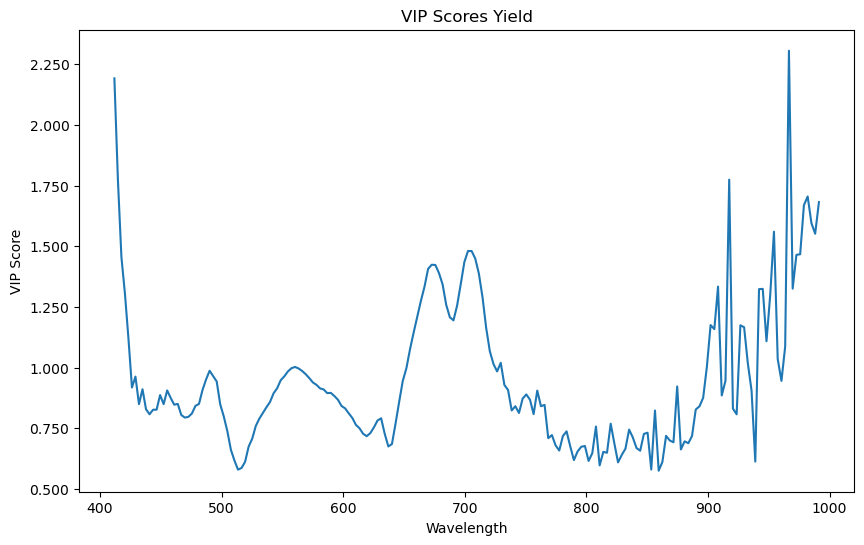

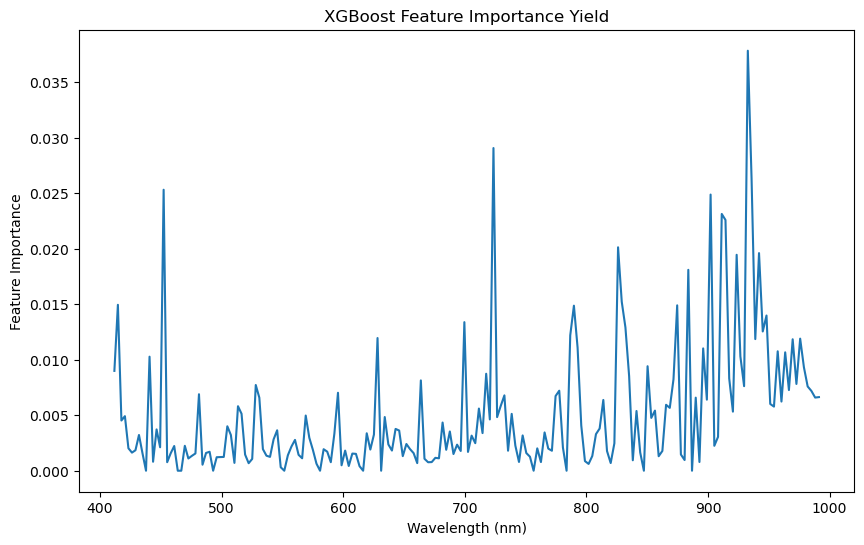


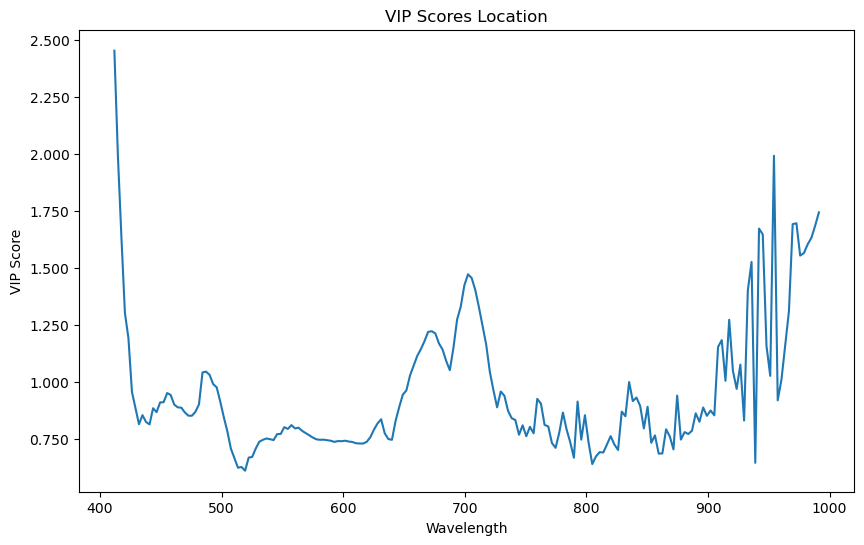

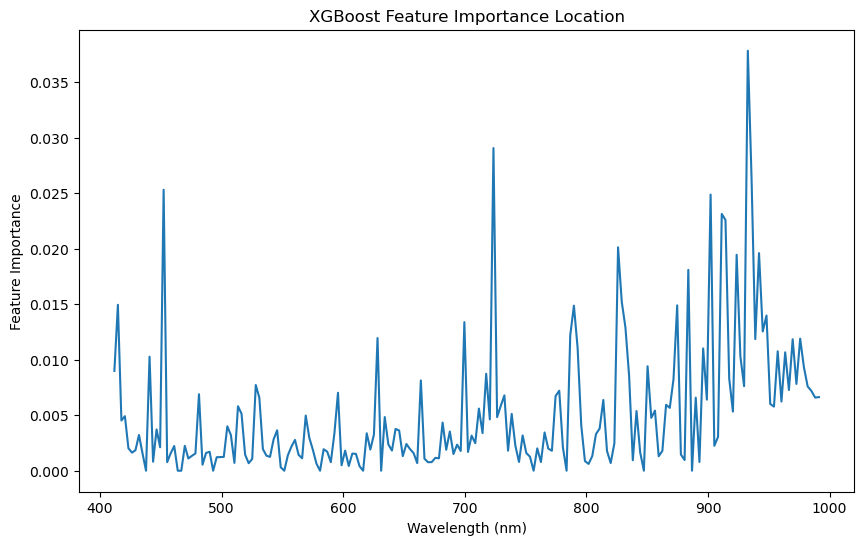


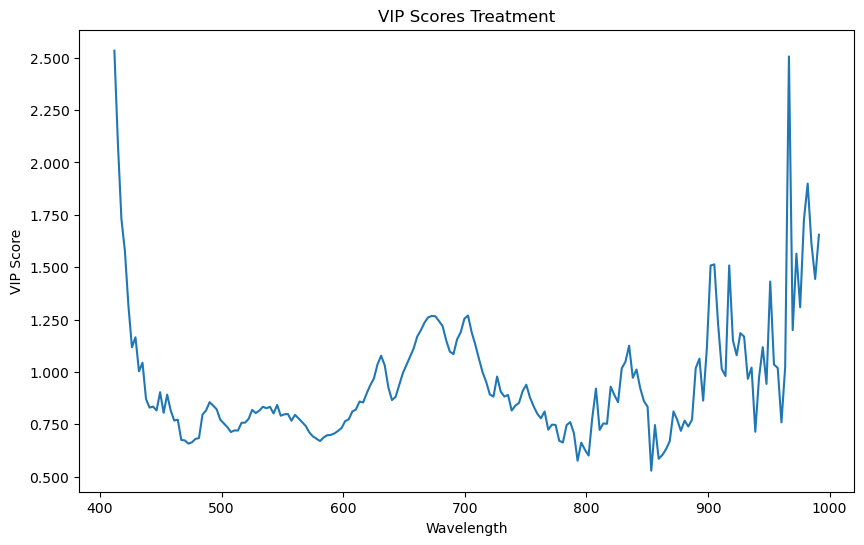

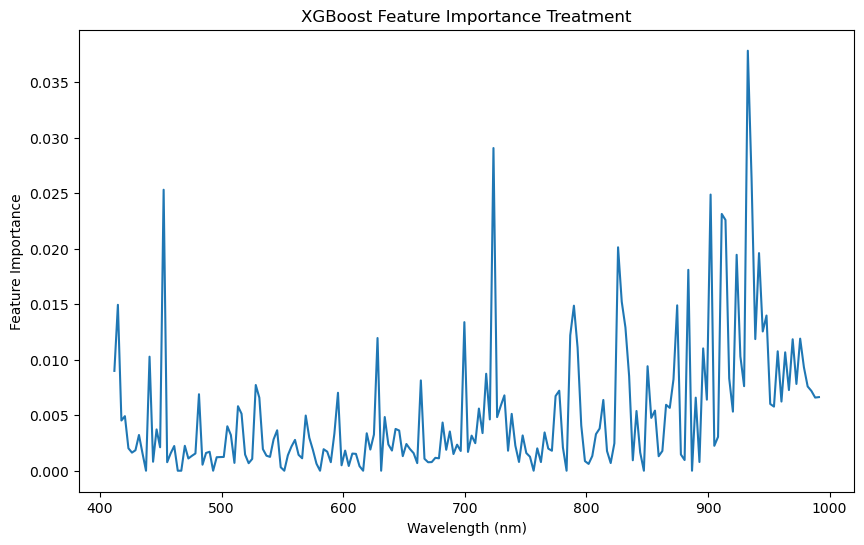


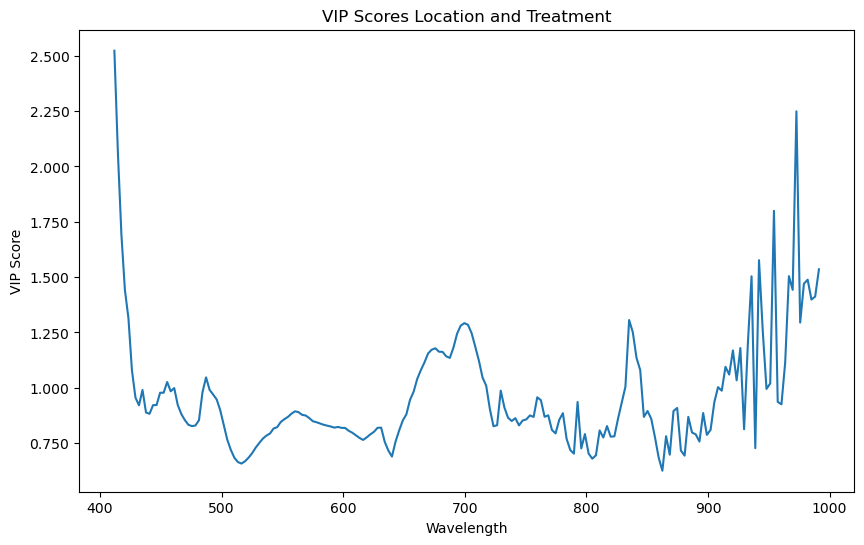

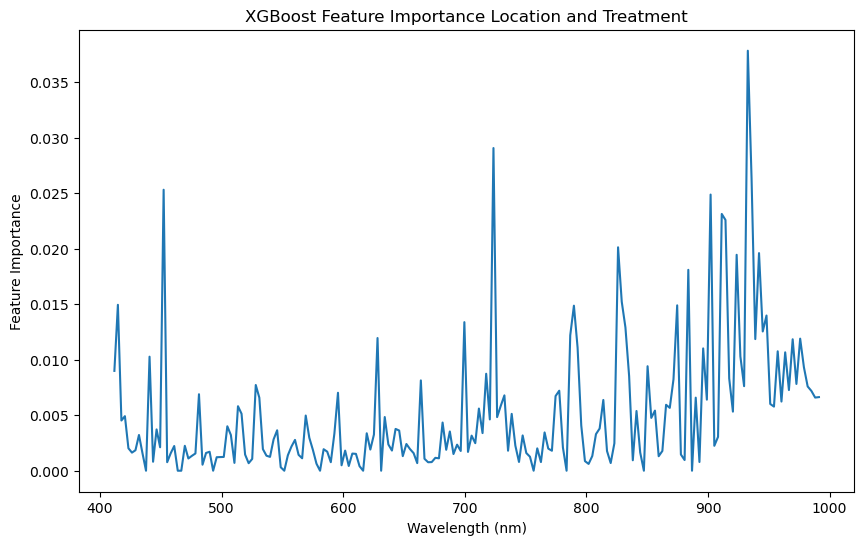

Supplement: Multimedia component 1 [file mmc1.docx]
